# Supplementary material for: Functional Spectrum of USP7 Pathogenic Variants in Hao-Fountain Syndrome: Insights into the Enzyme’s Activity, Stability, and Allosteric Modulation
Source: Proc Natl Acad Sci U S A. Author manuscript; Available in PMC 2025 Dec 5. (PMC12501124; doi:10.1073/pnas.2510252122)
Supplement: Supplementary Materials [file NIHMS2116533-supplement-Supplementary_Materials.pdf]

Figure S1

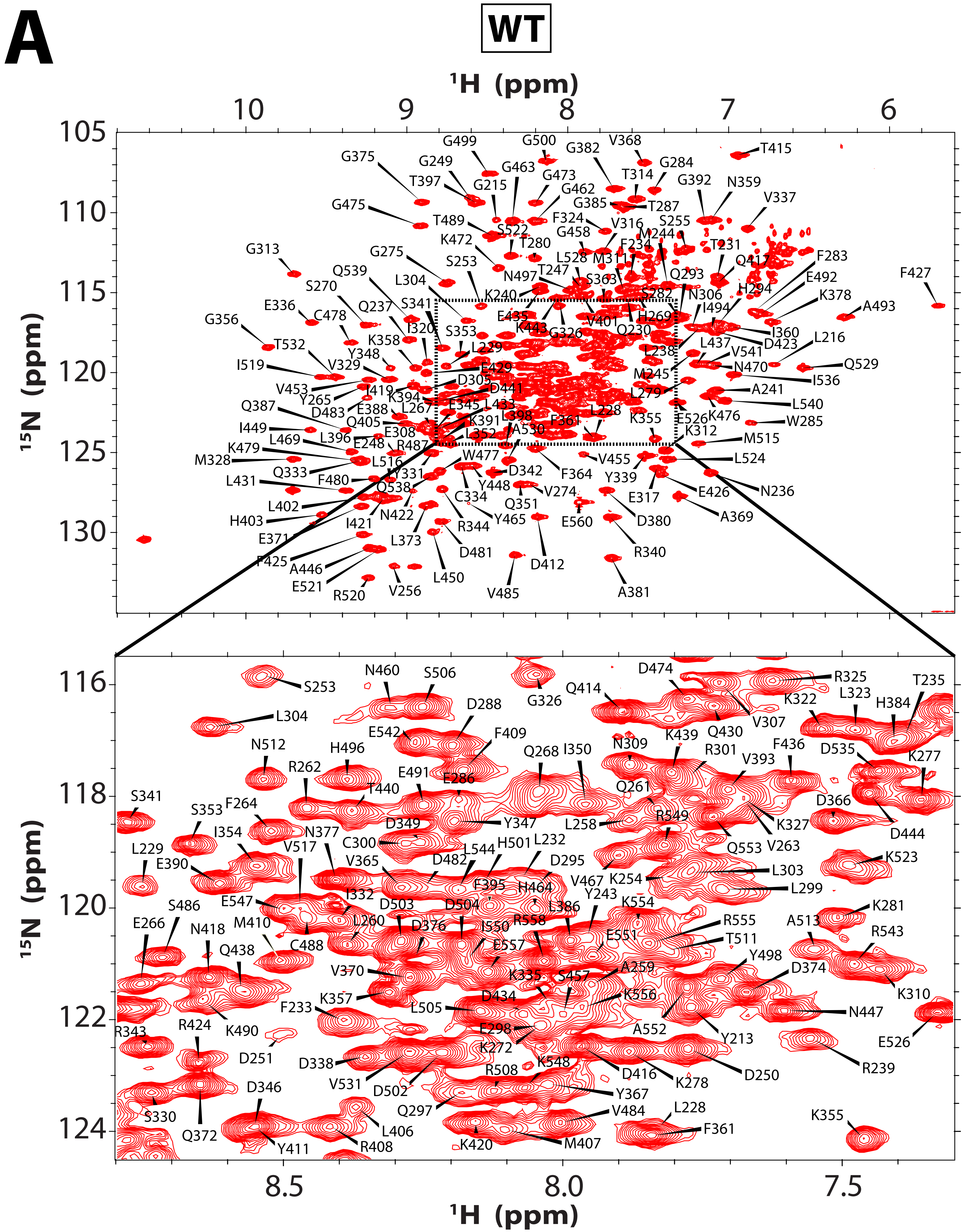

A
WT
1H (ppm)
10
9
8
7
6
105
110
115
120
125
130
15N (ppm)
G499
G500
G382
V368
T415
G375
G249
G463
G473
G284
T397
G215
G462
T314
G392
N359
G385
T287
V337
G475
T489
S522
F324
V316
S255
K472
T280
G458
M244
T231
G313
G275
L528
M311
F234
Q417
F283
S253
N497
T247
S363
Q293
Q539
L304
K240
S282
N306
H294
E492
S270
S341
E435
H269
I494
K378
A493
F427
E336
Q237
C478
K443
G326
V401
Q230
I360
G356
I320
S353
K358
D423
L216
T532
Y348
L229
L437
V541
L238
Q529
I519
V329
E429
N470
D305
M245
I536
V453
I419
D441
A241
Y265
K394
L279
Q387
D483
E388
L267
E345
L433
E526
K476
L540
I449
Q405
K391
L398
F361
L228
K355
W285
E308
A530
K312
L469
L396
E248
R487
L352
M515
K479
M328
L516
W477
V455
L524
Q333
Y331
D342
F364
Y339
E426
N236
L431
F480
Q538
Y448
V274
E317
C334
Q351
L402
N422
Y465
E560
D380
A369
H403
I421
R344
E371
L373
D481
R340
F425
A446
D412
L450
E521
V256
V485
A381
R520
S253
N460
S506
D474
R325
T235
G326
Q414
K322
L323
L304
D288
H384
V307
E542
Q430
F409
K439
R301
N512
H496
Q268
I350
N309
F436
D535
R262
V393
K277
E491
E286
T440
Q261
S341
D366
Y347
F264
L258
R549
K327
S353
D349
D444
I354
N377
C300
H501
L232
Q553
V263
L229
V517
L544
K523
E390
D482
D295
V365
L303
V467
K254
F395
H464
L299
Y243
S486
E547
I332
D503
D504
L386
K554
K281
E266
M410
L260
D376
I550
R558
R555
A513
N418
E551
R543
C488
T511
Q438
E557
Y498
V370
A259
D374
K335
S457
K357
K556
K310
D434
F233
L505
N447
R343
R424
K490
E298
A552
Y213
D251
E526
K272
D338
K548
D416
K278
D250
R239
R508
V531
D502
Y367
D346
Q297
V484
L228
K355
S330
L406
F361
Q372
Y411
R408
K420
M407
8.5
8.0
7.5
116
118
120
122
124
1H (ppm)
15N (ppm)

Figure S1

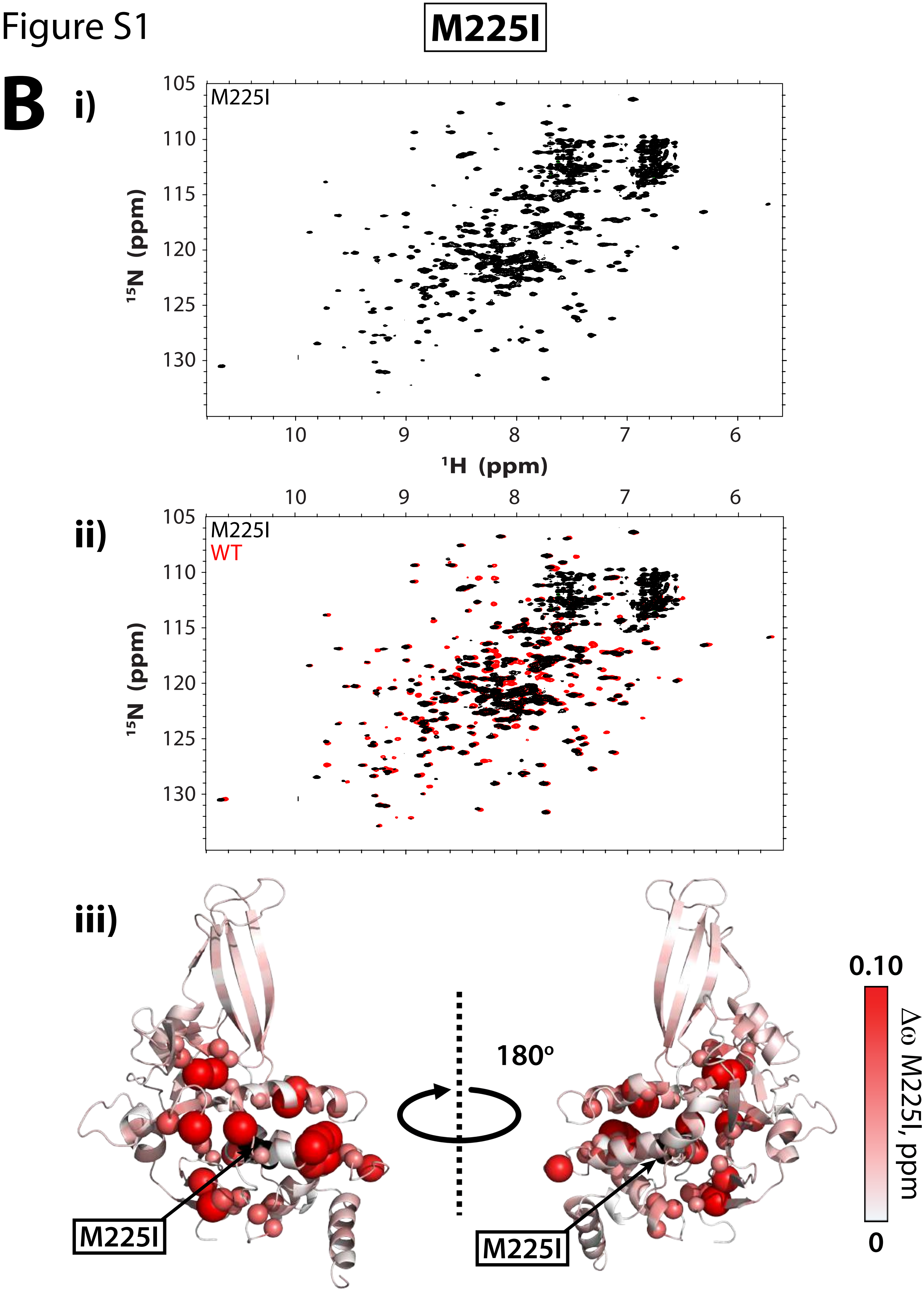
M225I
B
i)
M225I
105
110
115
120
125
130
¹⁵N (ppm)
10
9
8
7
6
¹H (ppm)
ii)
M225I
WT
iii)
180º
M225I
M225I
0.10
0
Δω M225I, ppm

Figure S1

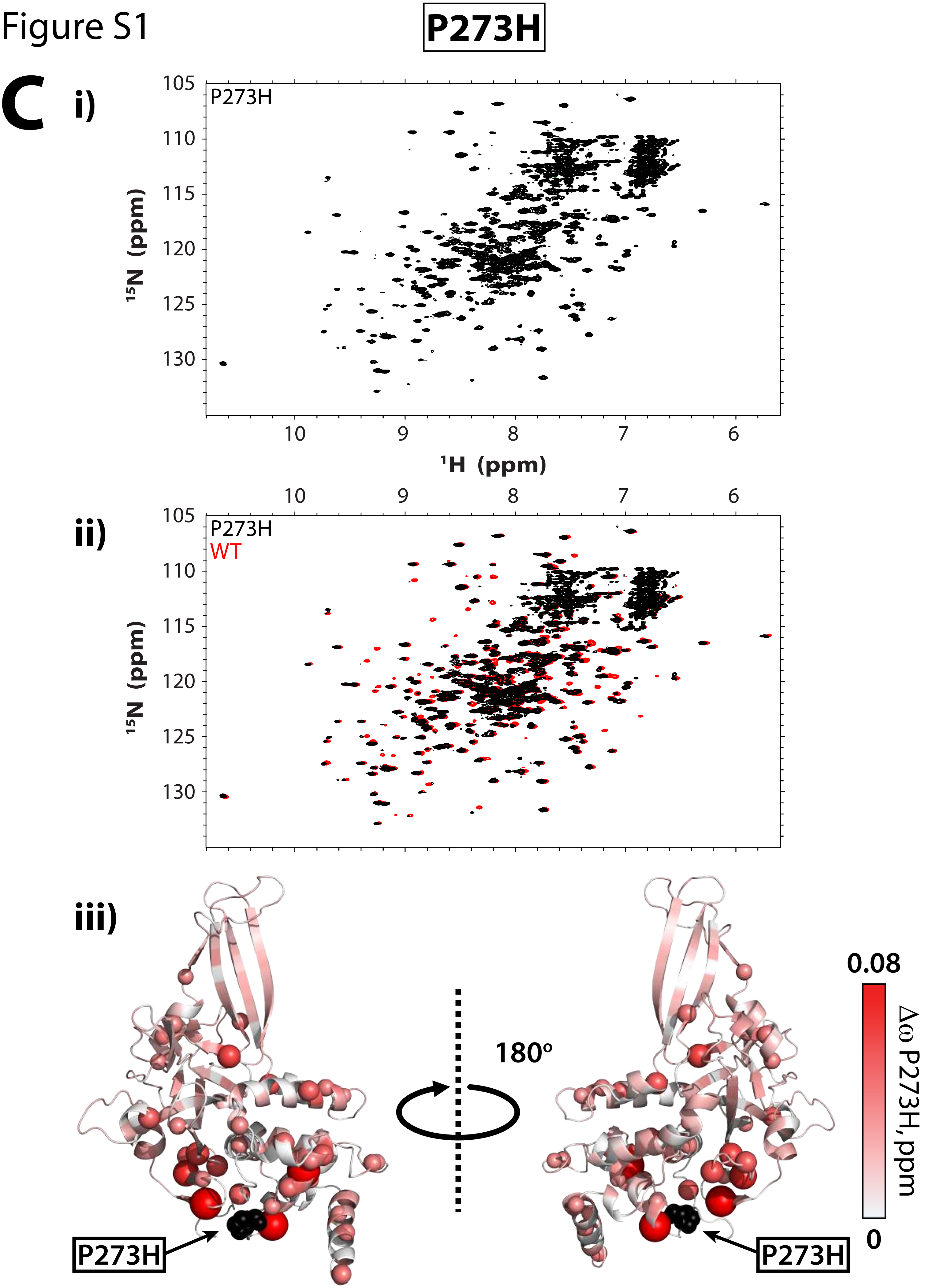
P273H
C
i)
P273H
105
110
115
120
125
130
15N (ppm)
10
9
8
7
6
1H (ppm)
ii)
P273H
WT
iii)
180°
0.08
0
Δω P273H, ppm
P273H
P273H

Figure S1

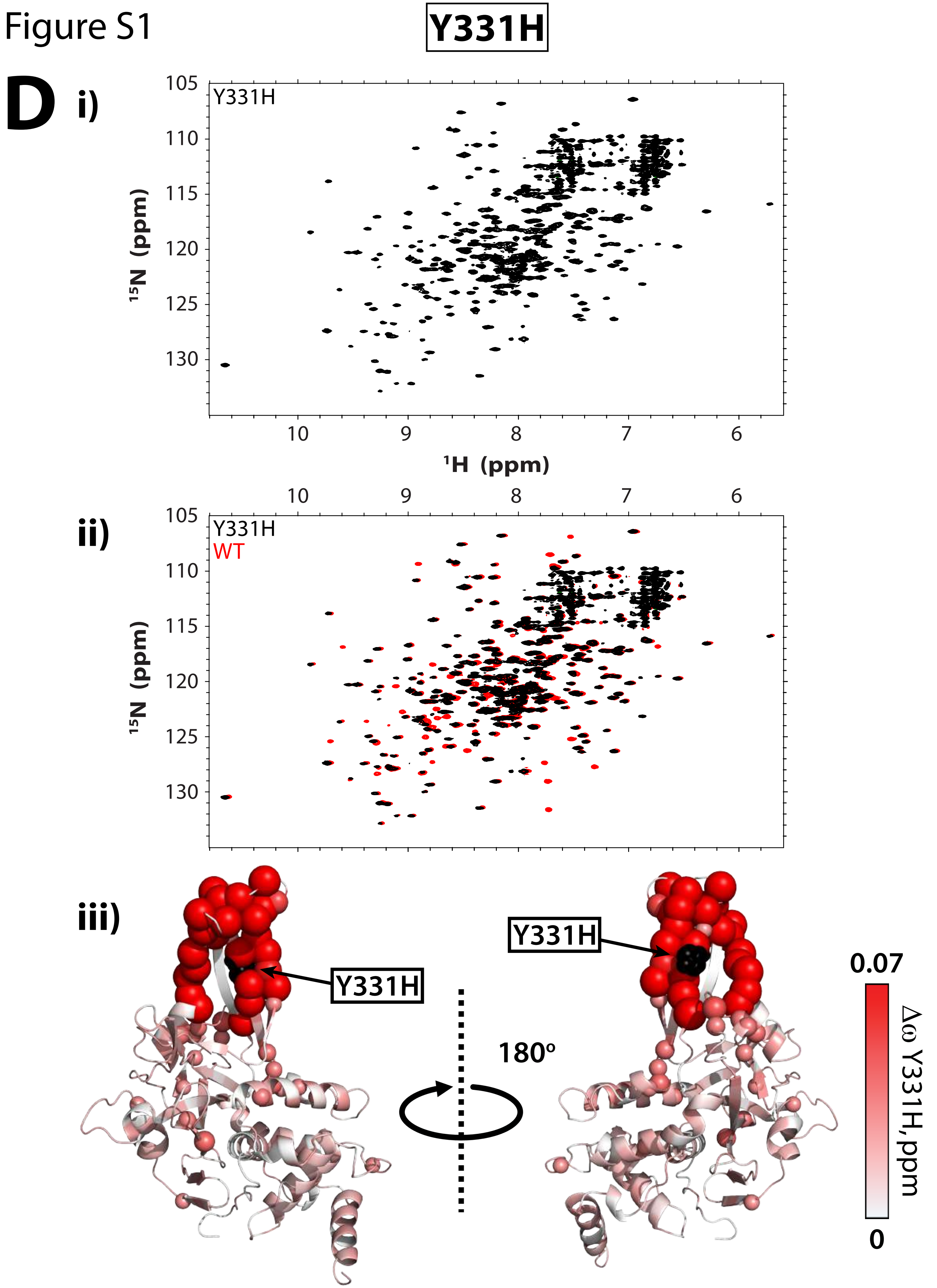

Y331H
D
i)
Y331H
105
110
115
120
125
130
^{15}N (ppm)
10
9
8
7
6
^{1}H (ppm)
ii)
Y331H
WT
iii)
Y331H
Y331H
180º
0.07
0
Δω Y331H, ppm

Figure S1

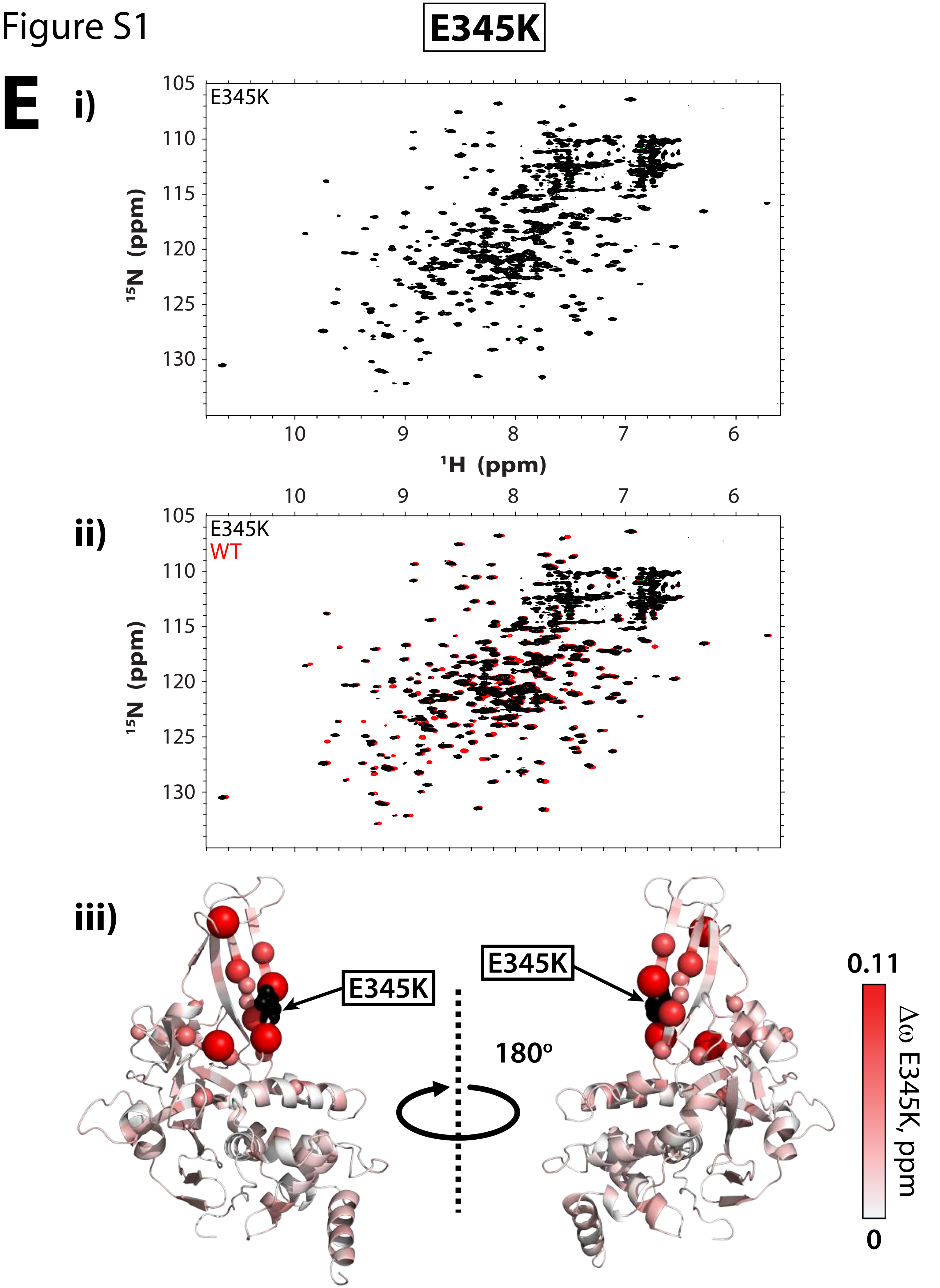
E345K
E
i)
E345K
105
110
115
120
125
130
15N (ppm)
10
9
8
7
6
1H (ppm)
ii)
E345K
WT
iii)
E345K
E345K
180°
0.11
Δω E345K, ppm
0

Figure S1

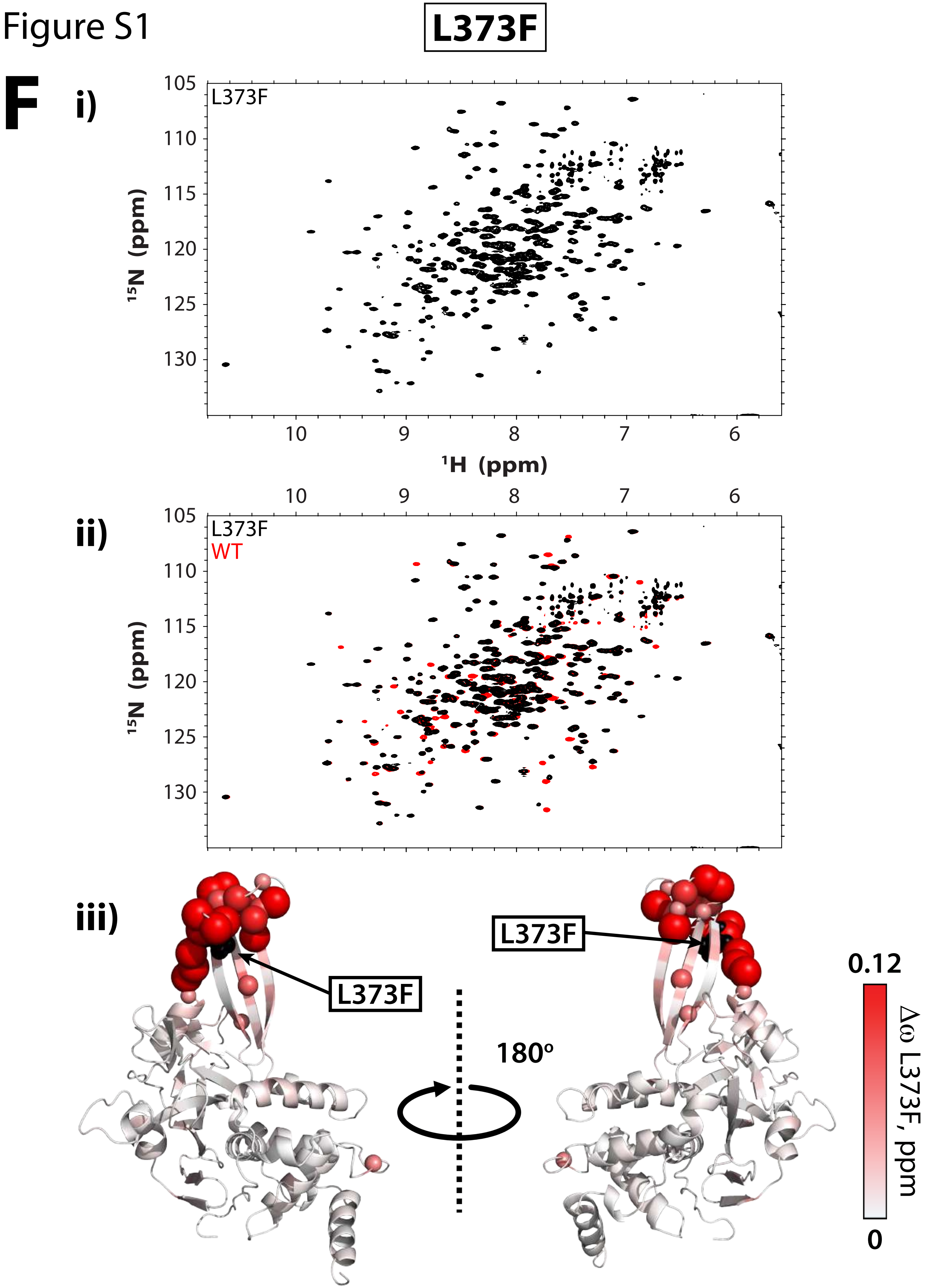
L373F
F
i)
L373F
105
110
115
120
125
130
^{15}N (ppm)
10
9
8
7
6
^{1}H (ppm)
ii)
L373F
WT
iii)
L373F
L373F
180°
0.12
0
Δω L373F, ppm

Figure S1

G392A

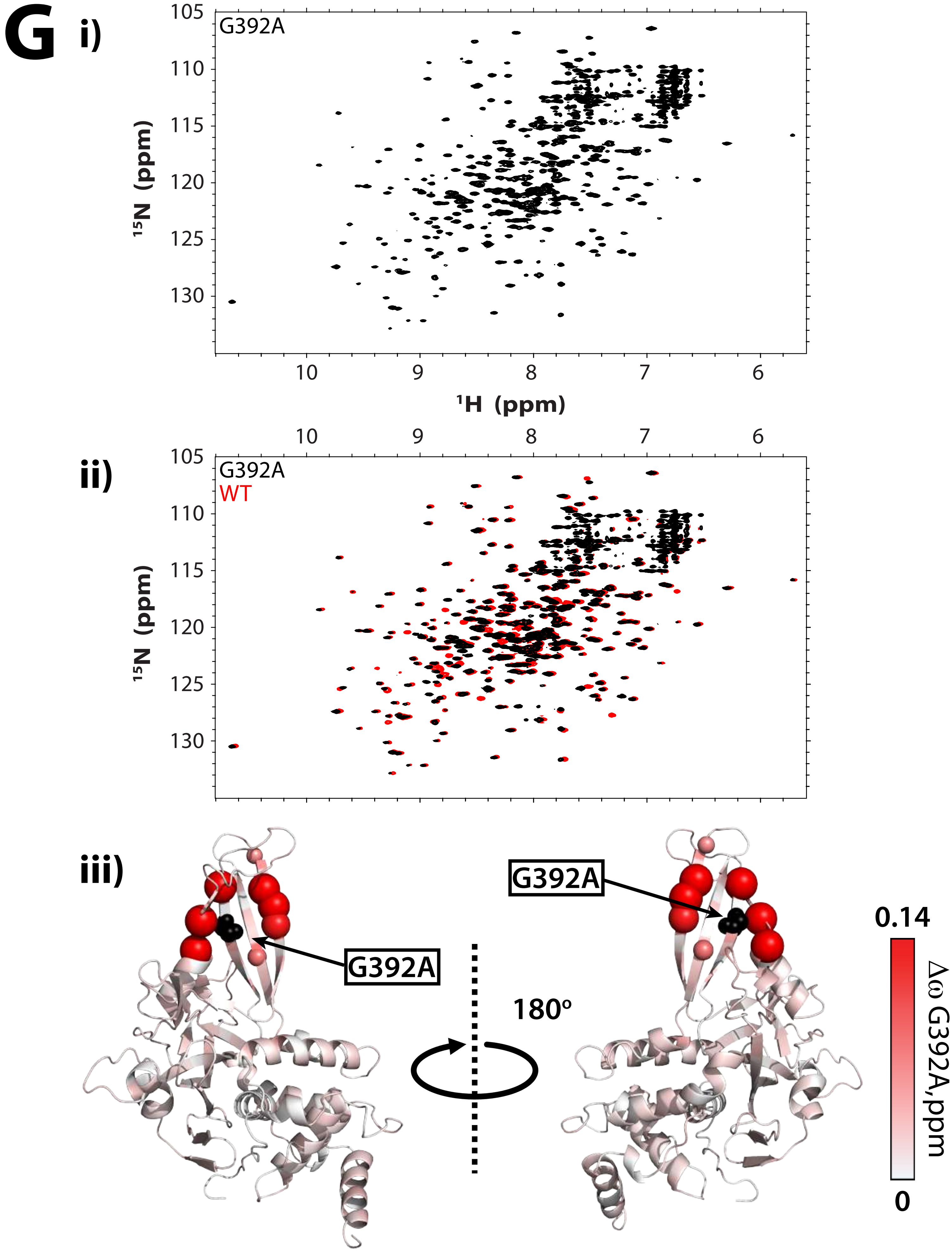

Figure S1

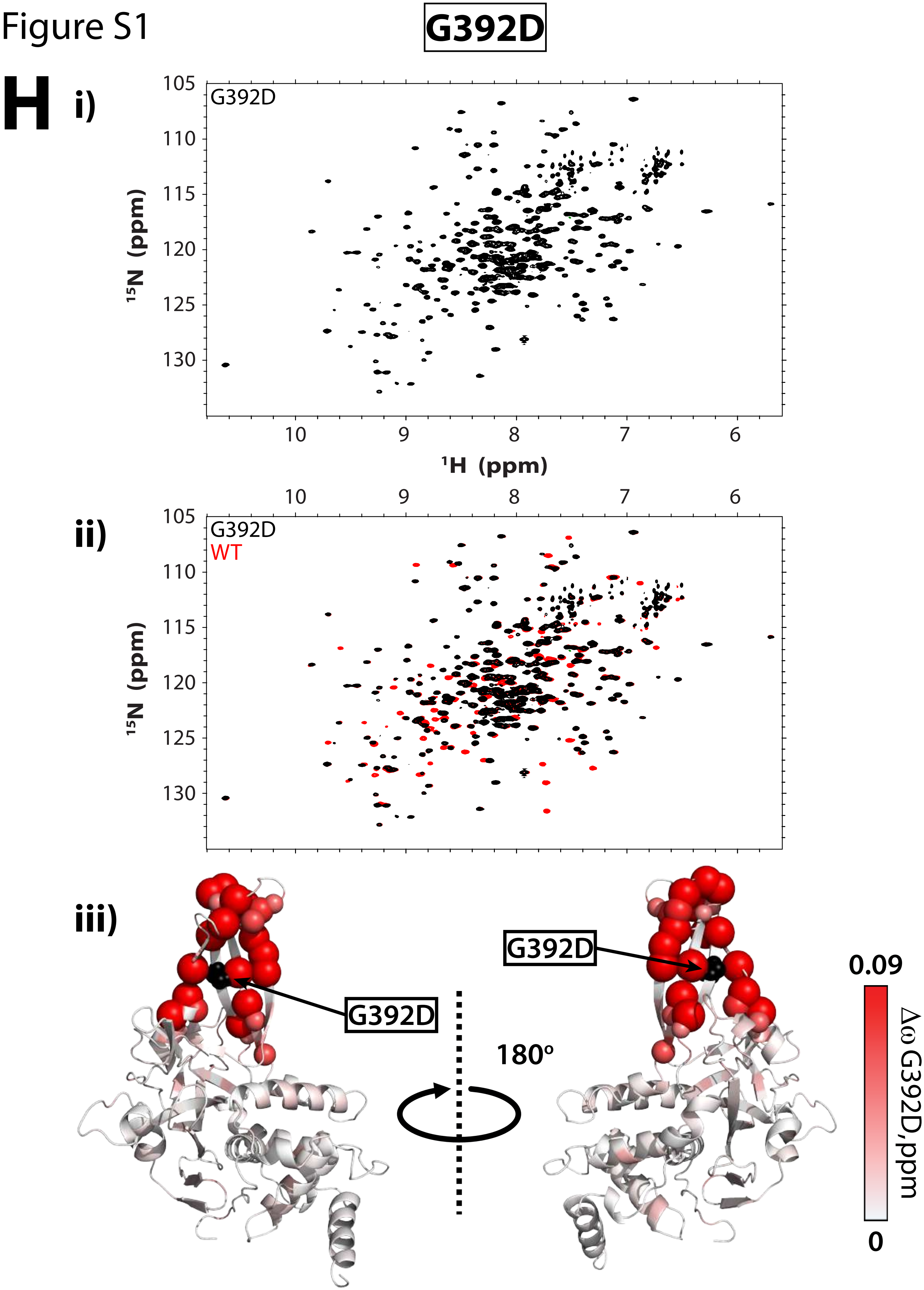
G392D
H
i)
G392D
105
110
115
120
125
130
^{15}N (ppm)
10
9
8
7
6
^{1}H (ppm)
ii)
G392D
WT
iii)
G392D
G392D
180°
0.09
0
Δω G392D,ppm

Figure S1

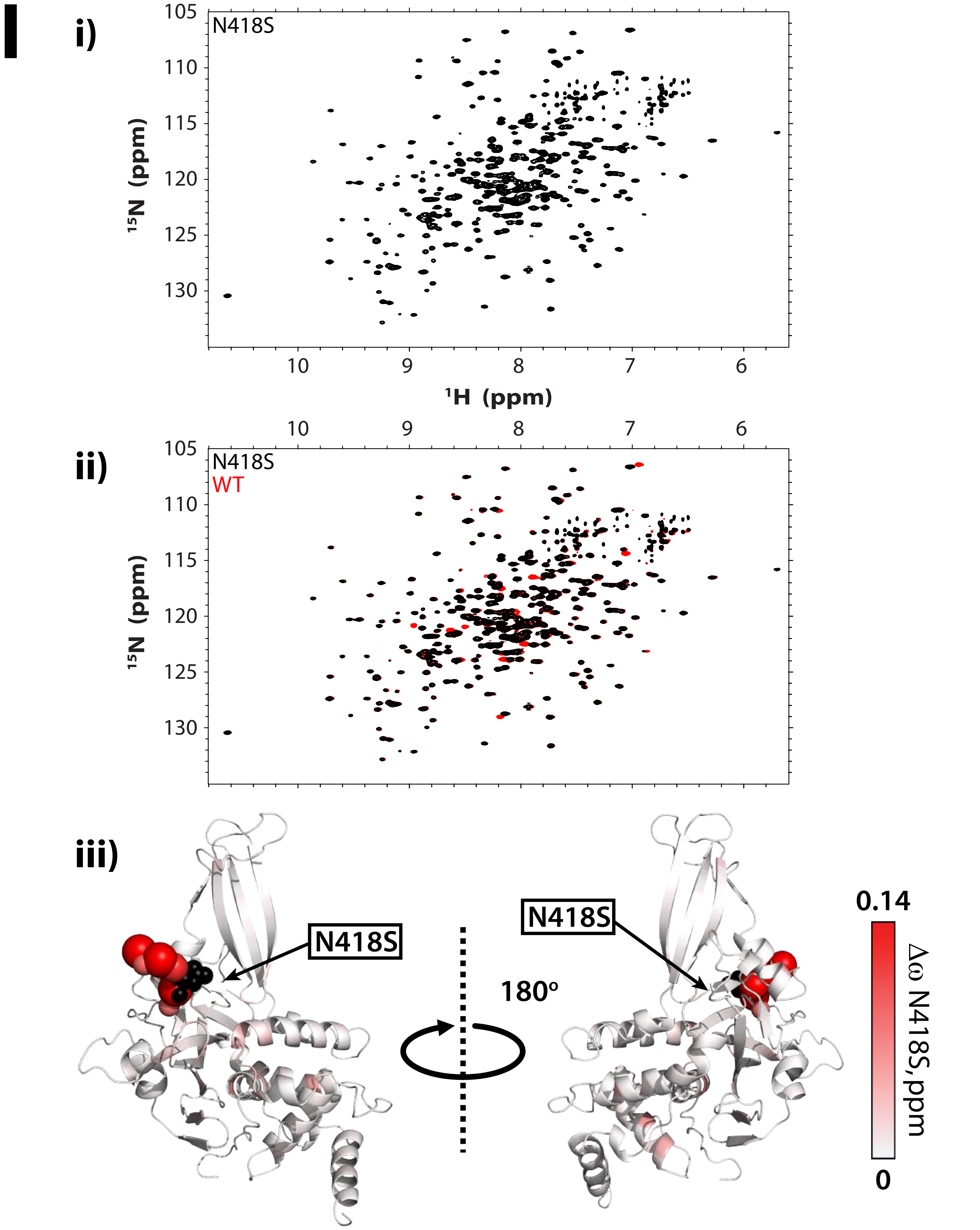

N418S
i)
N418S
105
110
115
120
125
130
^{15}N (ppm)
10
9
8
7
6
^{1}H (ppm)
ii)
N418S
WT
iii)
N418S
180°
N418S
0.14
0
Δω N418S, ppm

Figure S1

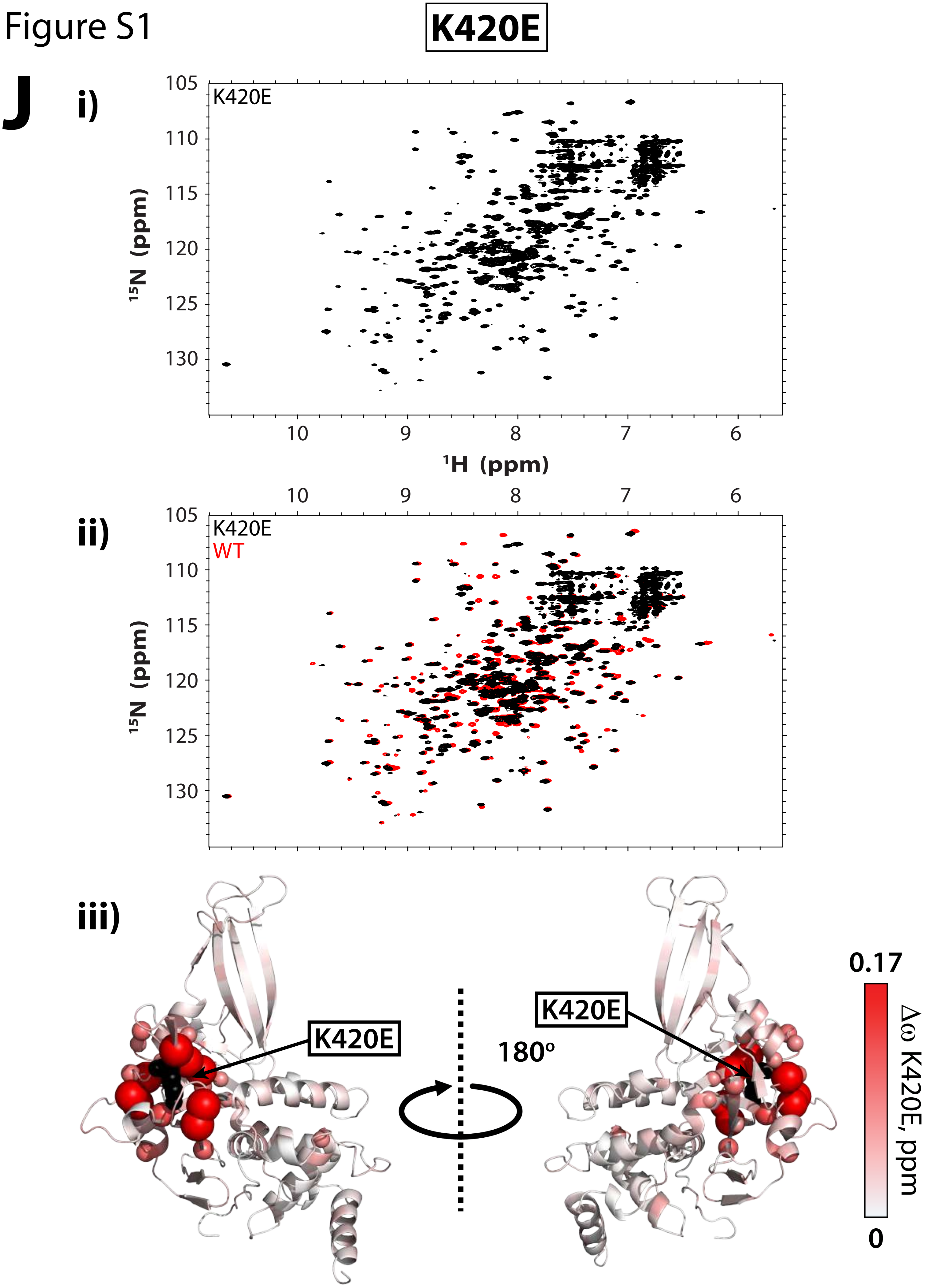
K420E
J
i)
K420E
105
110
115
120
125
130
^{15}N (ppm)
10
9
8
7
6
^{1}H (ppm)
ii)
K420E
WT
iii)
K420E
180°
K420E
0.17
0
Δω K420E, ppm

Figure S1

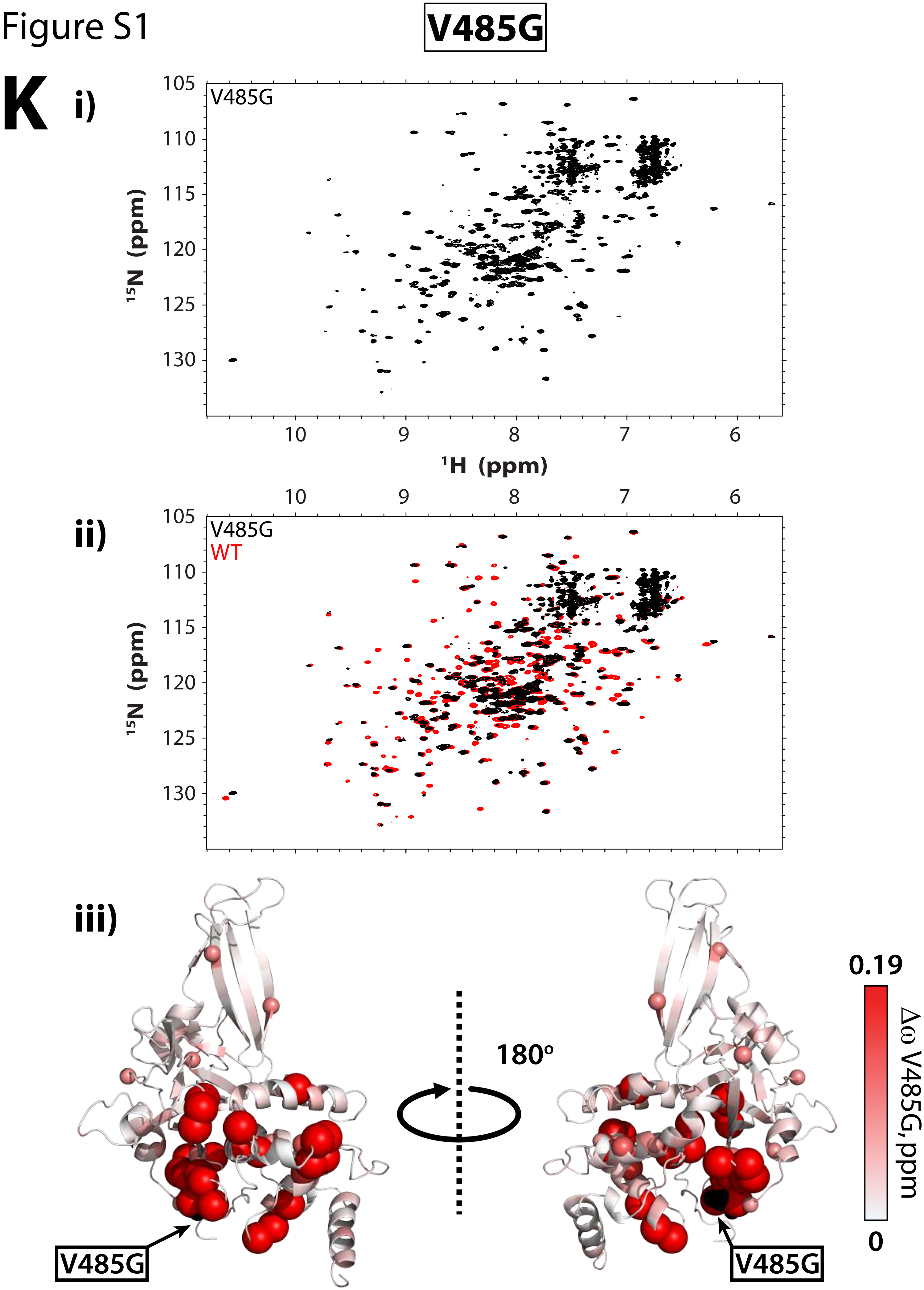

V485G
K
i)
V485G
105
110
115
120
125
130
15N (ppm)
10
9
8
7
6
1H (ppm)
ii)
V485G
WT
iii)
180º
0.19
0
Δω V485G,ppm
V485G
V485G

Figure S1. NMR spectra of USP7 variants associated with Hao-Fountain syndrome.
2D ^{15}N TROSY spectra of ^{15}N-labeled USP7 catalytic domain and its Hao-Fountain syndrome variants. **A.** Sequence-specific resonance assignments are indicated for the WT (BMRB ID: 51188). The inset shows an enlarged area of the spectrum that corresponds to the region outlined by dashed lines. **B-K. (i)** Individual spectra are shown in black for each variant. **(ii)** The WT spectrum (red) is overlayed with each variant (black). **(iii)** Chemical shift perturbations ($\Delta\omega$ mut) between WT and variant spectra are mapped onto the structure of the USP7 catalytic domain in two orientations (PDB ID: 5JTV) (38), to highlight structural changes associated with the mutation. Individual mutations are indicated as black spheres and labeled. Regions with higher $\Delta\omega$ are shown in more saturated red. Mapping was based on each variant's maximal $\Delta\omega$, with residues exceeding 40% of that maximum displayed as spheres. Residues missing in the spectrum of a mutant were assigned the maximal CSP value.

Figure S2

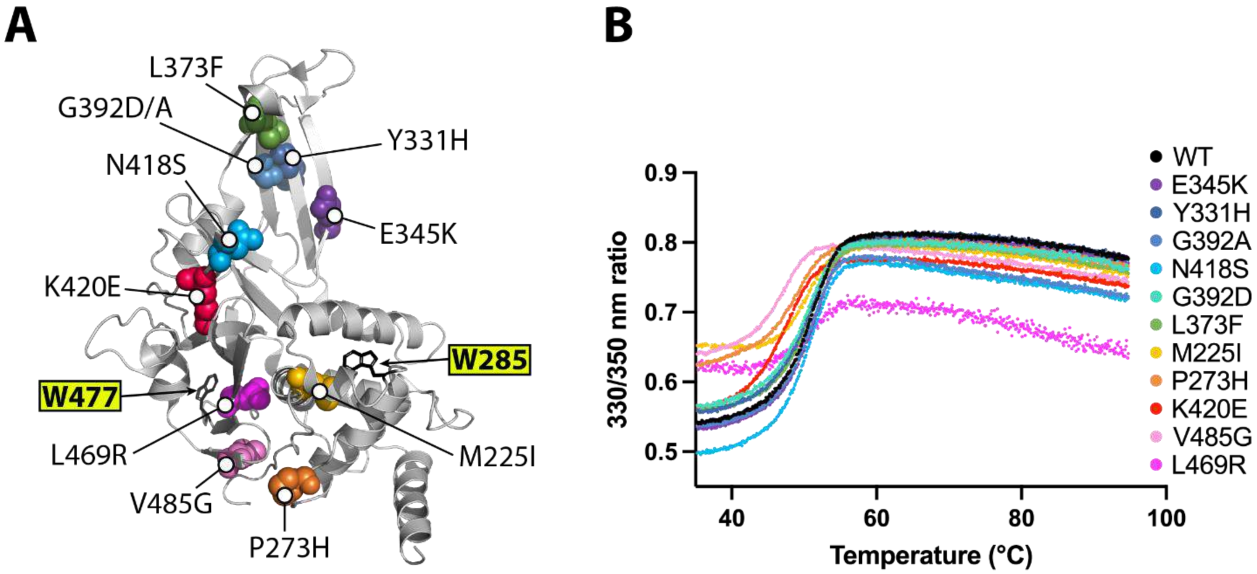

Figure S2. Effect of Hao-Fountain syndrome variants on USP7 stability.
A. Hao-Fountain variants mapped as spheres on the structure of the USP7 catalytic domain (PDB ID: 5JTV) (38), as in **Figure 1D**. Mutations are color-coded based on their individual thermostability. The two tryptophans are highlighted in yellow. **B.** Thermostability assays measuring the change in tryptophan fluorescence (330/350 nm ratio) as a function of temperature. The 330 nm signal reflects buried tryptophan fluorescence, while 350 nm corresponds to solvent-exposed tryptophan. The thermal melting curves are color-coded as in **S2A**.

Figure S3

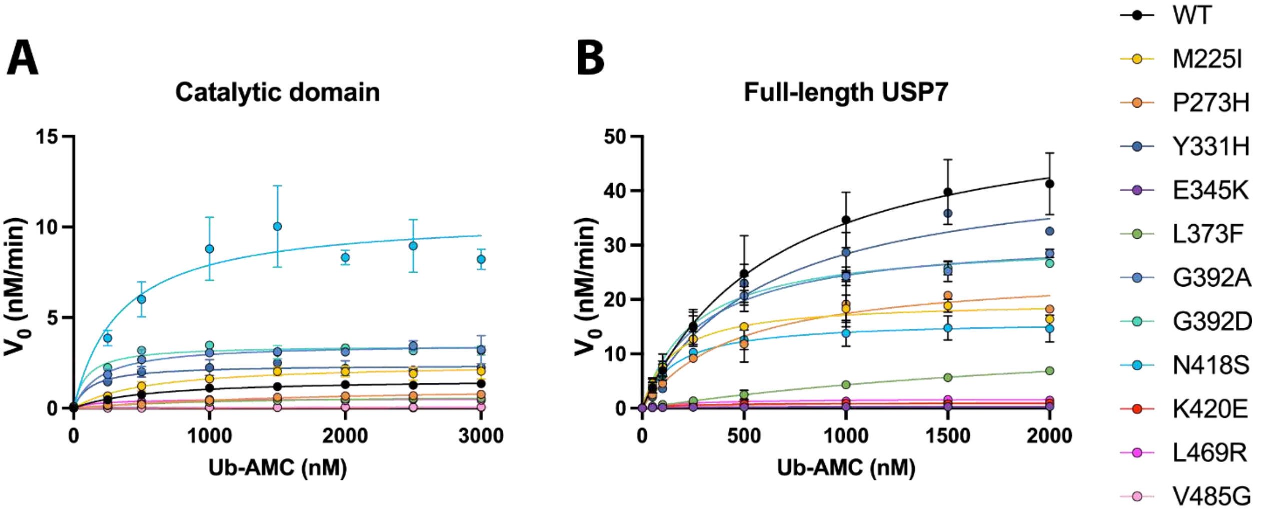

Figure S3. Effect of Hao-Fountain syndrome variants on USP7 activity.
Michaelis-Menten plots of the initial velocity (V_0) as a function of ubiquitin-AMC concentration shown for (**A**) USP7 catalytic domain and its mutants and (**B)** FL-USP7 and its mutants.

Figure S4

A

^{15}N USP7 (WT) : Ubiquitin

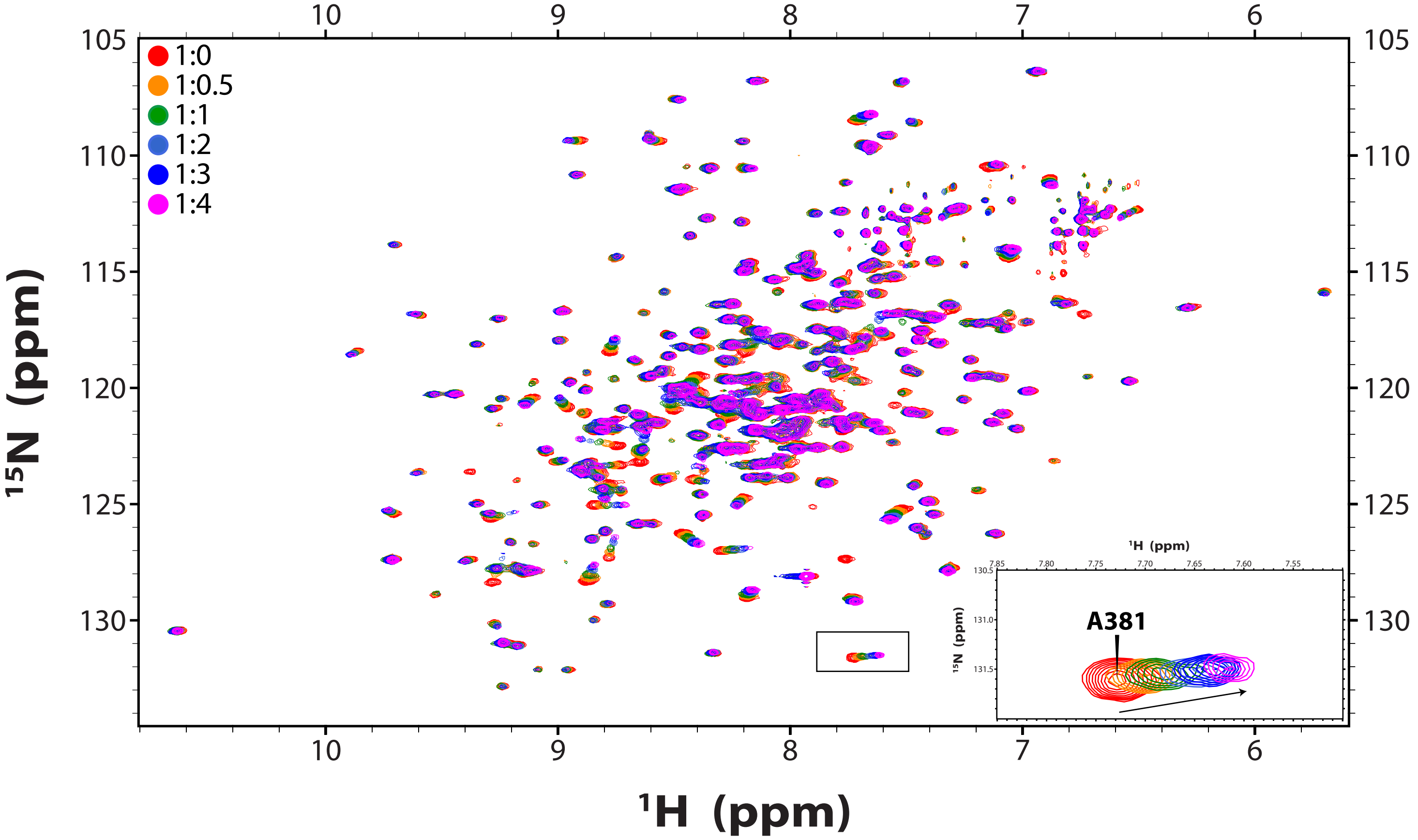

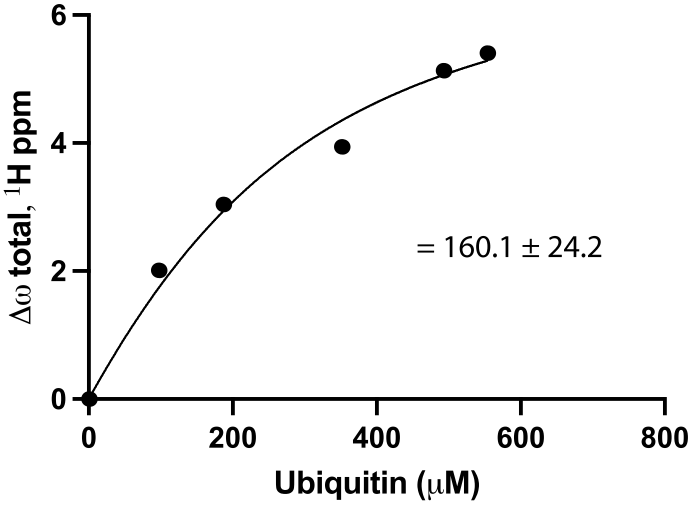

Figure S4

B

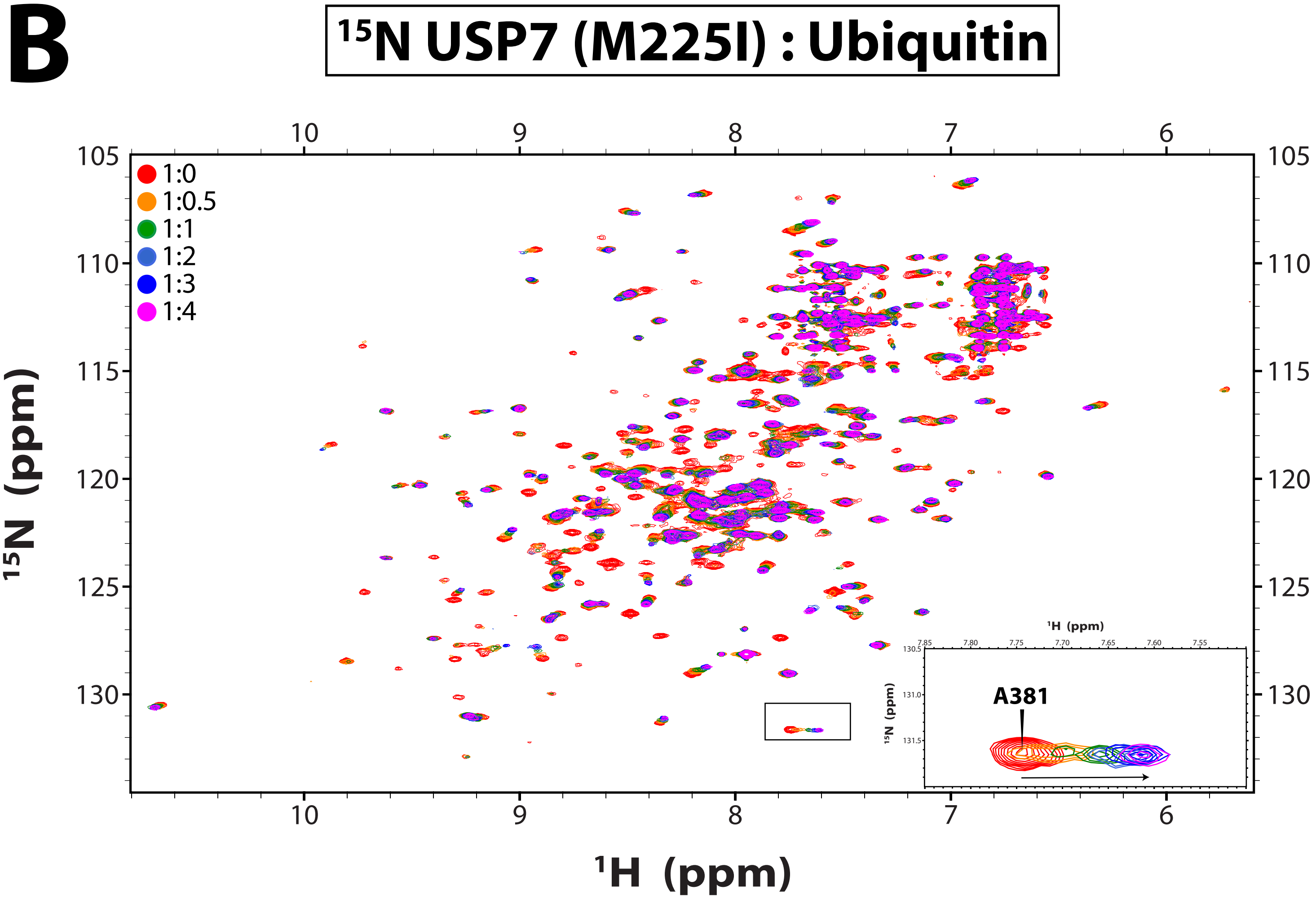

^{15}N USP7 (M225I) : Ubiquitin
1:0
1:0.5
1:1
1:2
1:3
1:4
^{15}N (ppm)
^{1}H (ppm)
A381
105
110
115
120
125
130
10
9
8
7
6

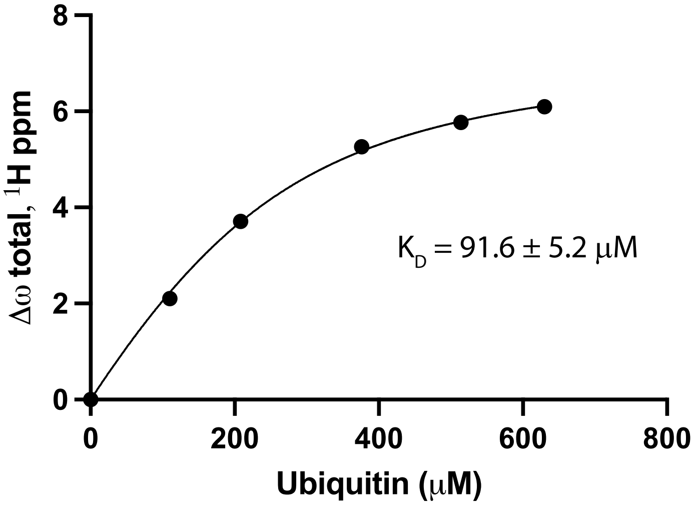

Δω total, ^{1}H ppm
Ubiquitin (μM)
K_D = 91.6 ± 5.2 μM
0
2
4
6
8
200
400
600
800

Figure S4

C

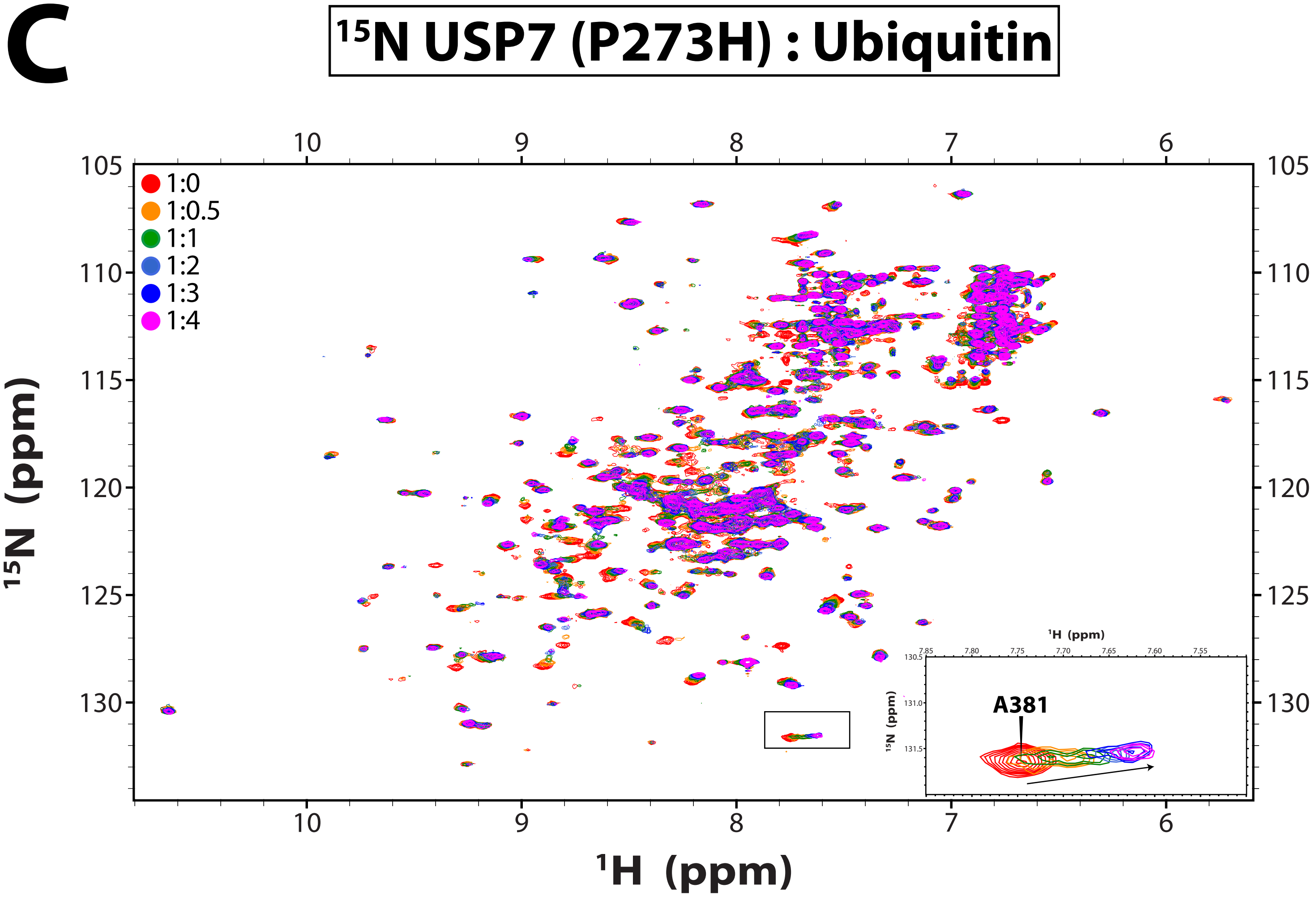
15N USP7 (P273H) : Ubiquitin
1:0
1:0.5
1:1
1:2
1:3
1:4
A381
15N (ppm)
1H (ppm)

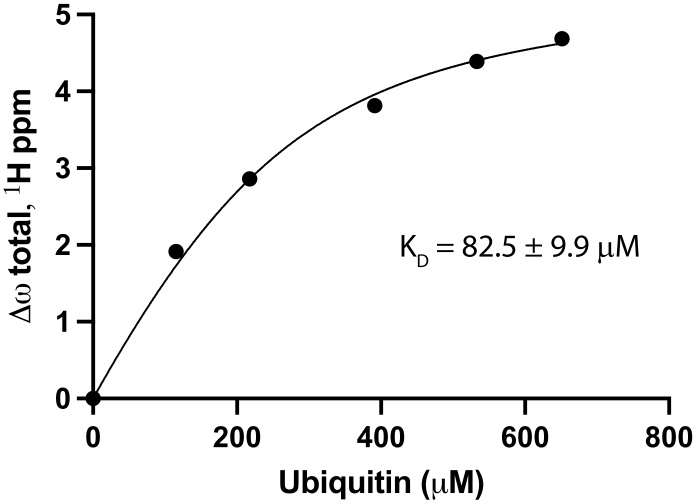
Δω total, 1H ppm
Ubiquitin (μM)
K_D = 82.5 ± 9.9 μM

Figure S4

D

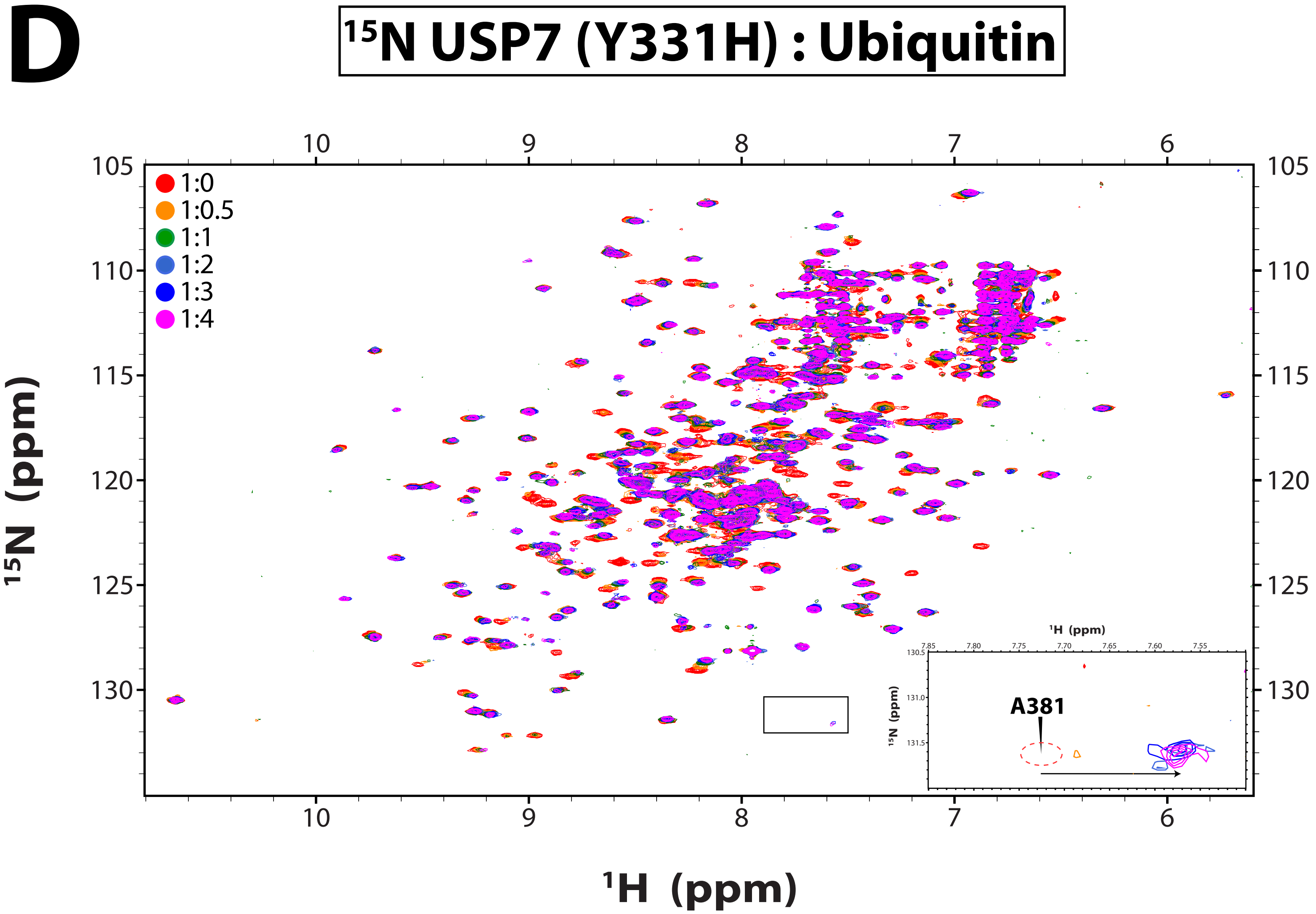

15N USP7 (Y331H) : Ubiquitin
1:0
1:0.5
1:1
1:2
1:3
1:4
A381
15N (ppm)
1H (ppm)
10
9
8
7
6
105
110
115
120
125
130

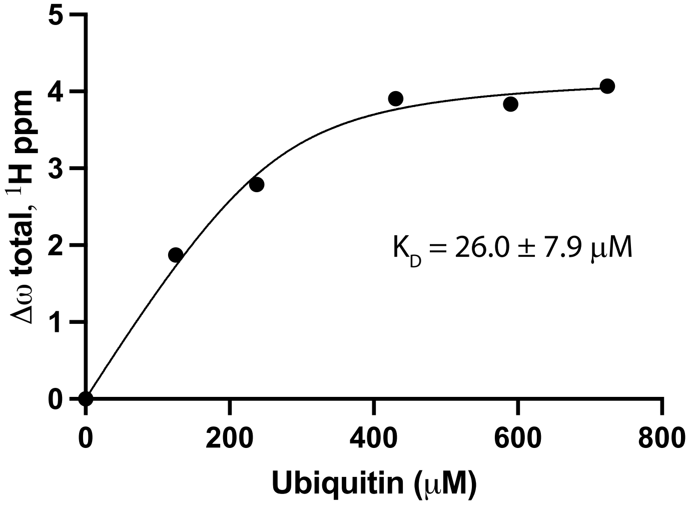

Δω total, 1H ppm
Ubiquitin (μM)
K_D = 26.0 ± 7.9 μM
0
200
400
600
800
0
1
2
3
4
5

Figure S4

E

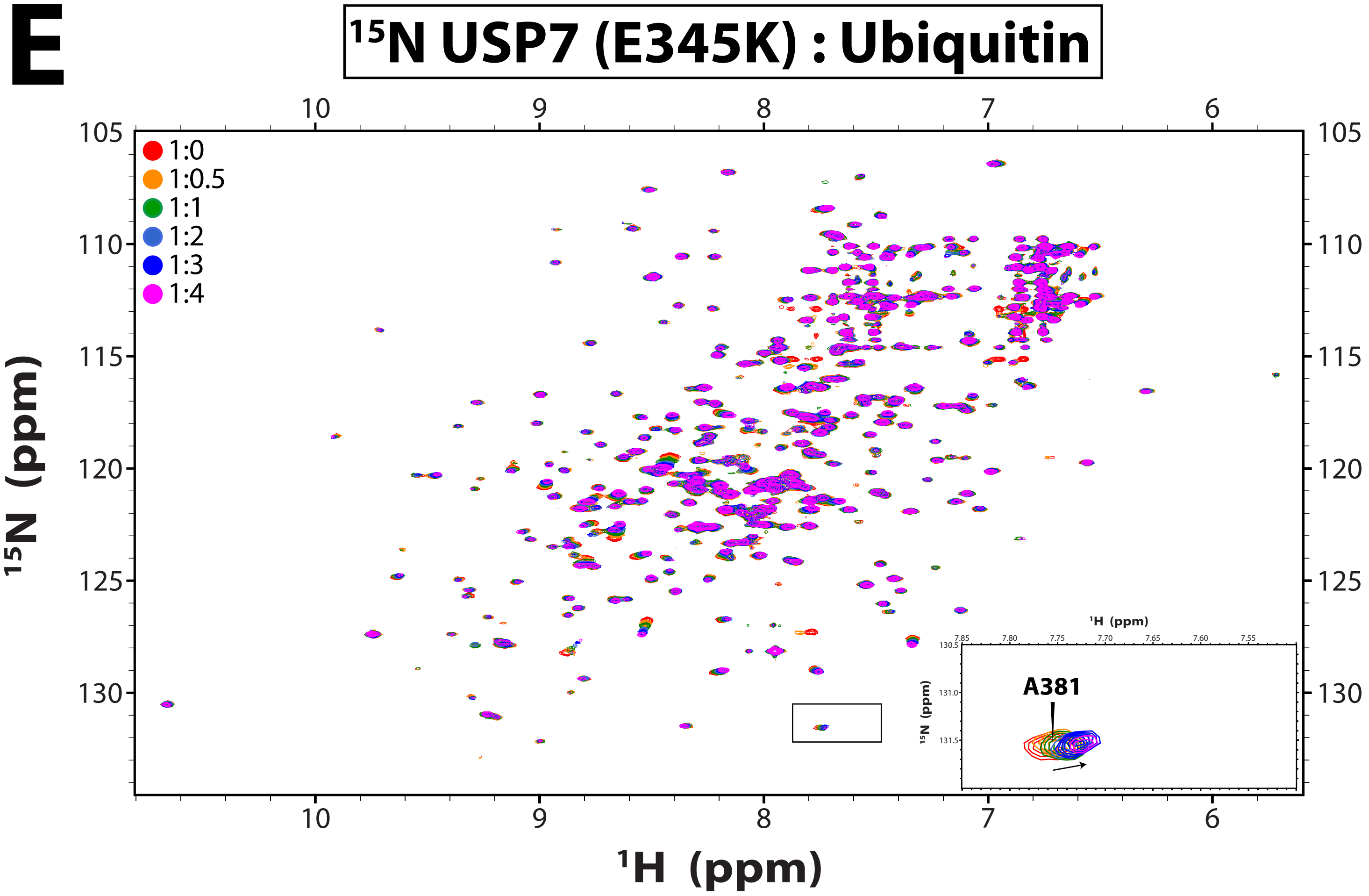

15N USP7 (E345K) : Ubiquitin
1:0
1:0.5
1:1
1:2
1:3
1:4
A381
1H (ppm)
15N (ppm)

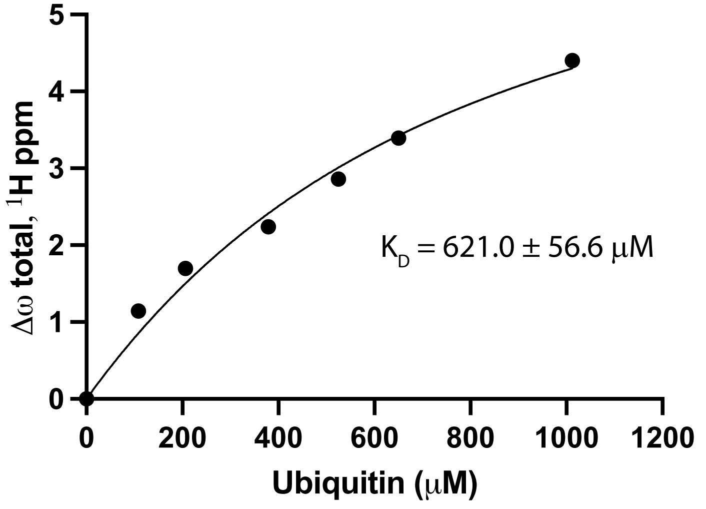

KD = 621.0 ± 56.6 μM
Δω total, 1H ppm
Ubiquitin (μM)

Figure S4

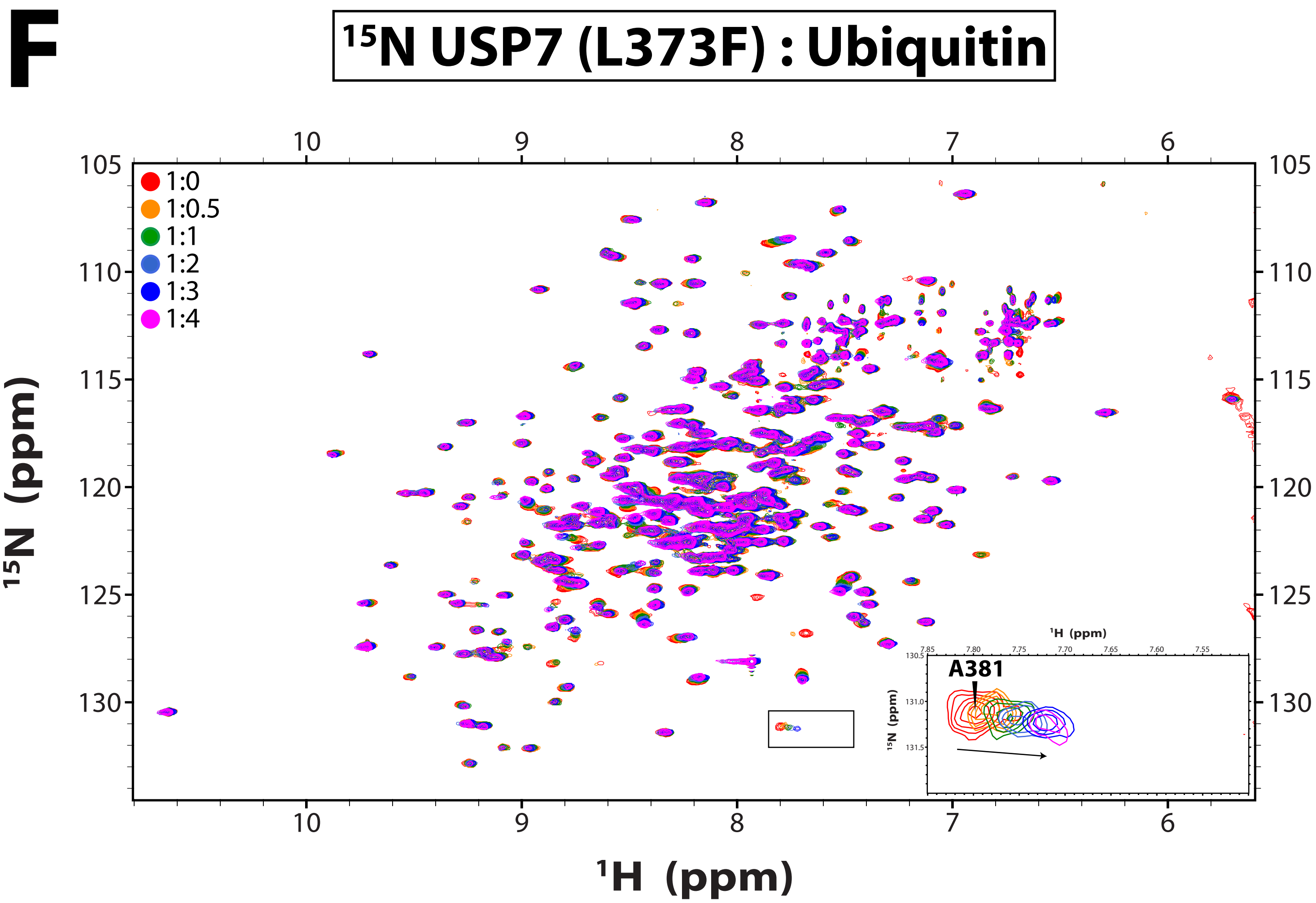
F
15N USP7 (L373F) : Ubiquitin
1:0
1:0.5
1:1
1:2
1:3
1:4
A381
15N (ppm)
1H (ppm)

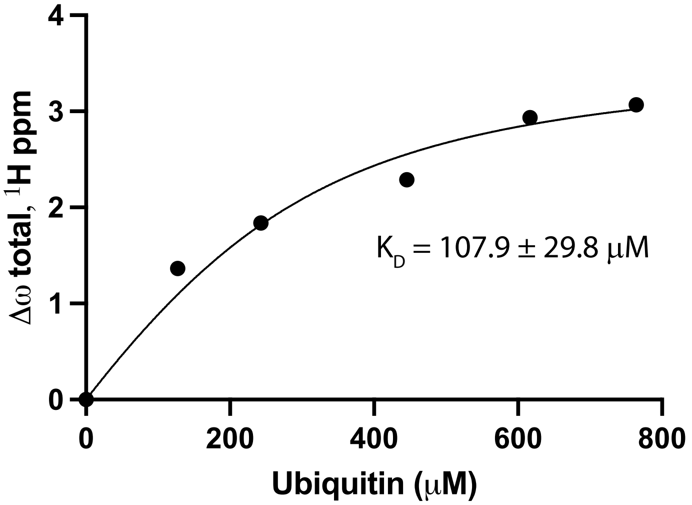
Δω total, 1H ppm
K$_D$ = 107.9 ± 29.8 μM
Ubiquitin (μM)

Figure S4

G

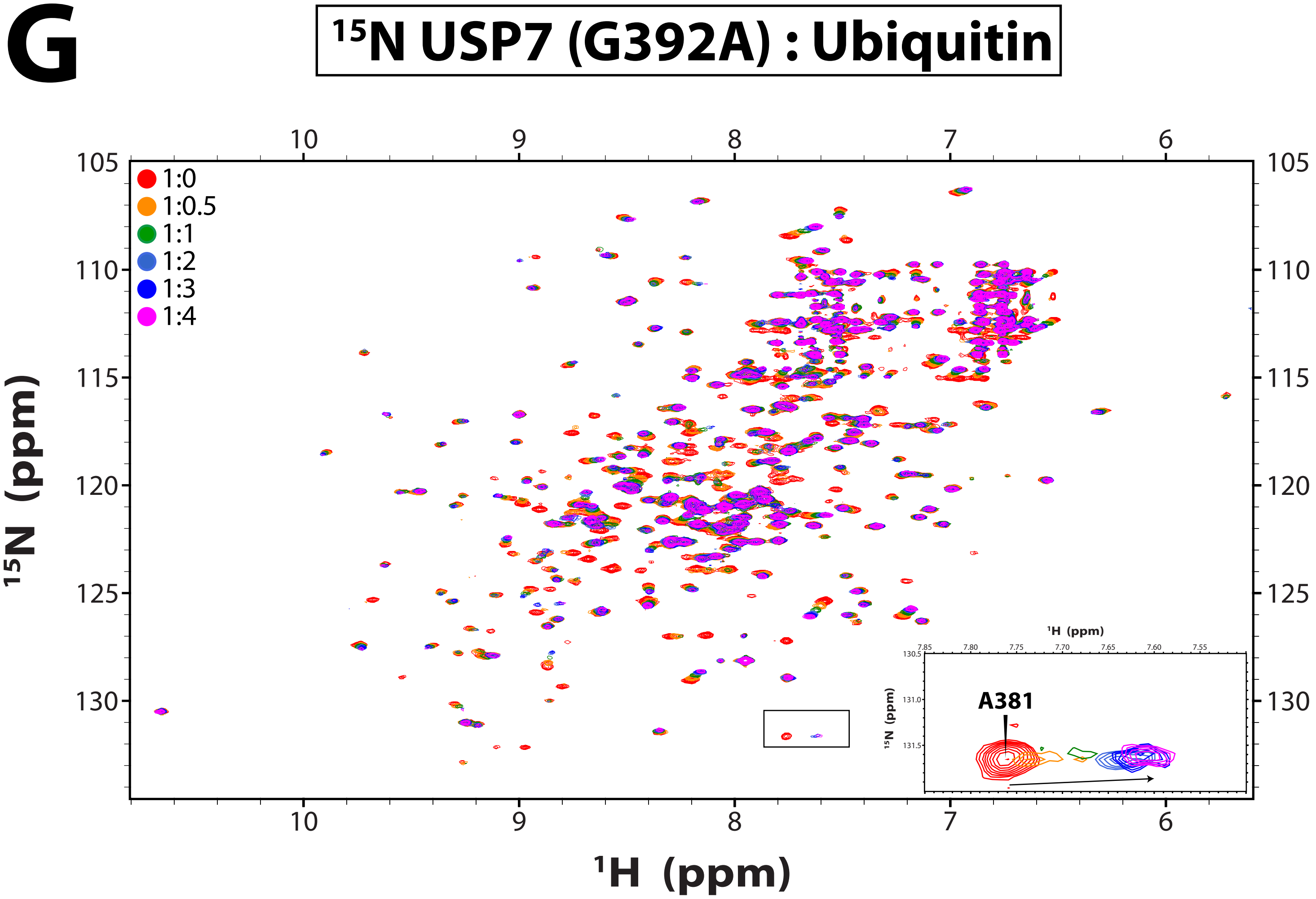

^{15}N USP7 (G392A) : Ubiquitin
1:0
1:0.5
1:1
1:2
1:3
1:4
^{15}N (ppm)
^{1}H (ppm)
A381

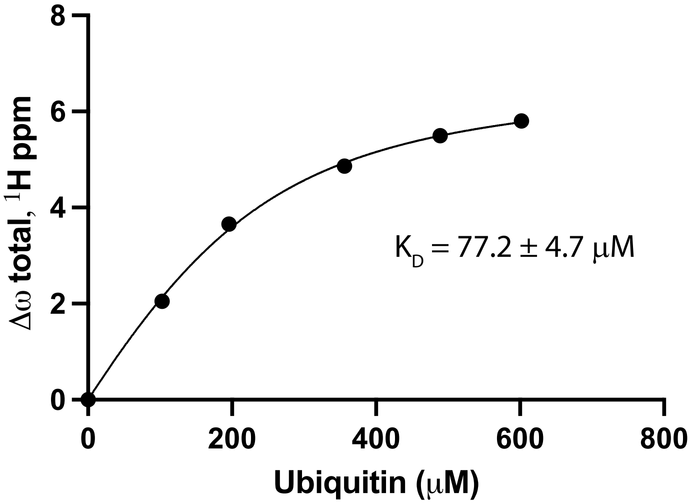

$\Delta\omega$ total, ^{1}H ppm
Ubiquitin (μM)
K_D = 77.2 ± 4.7 μM

Figure S4

H

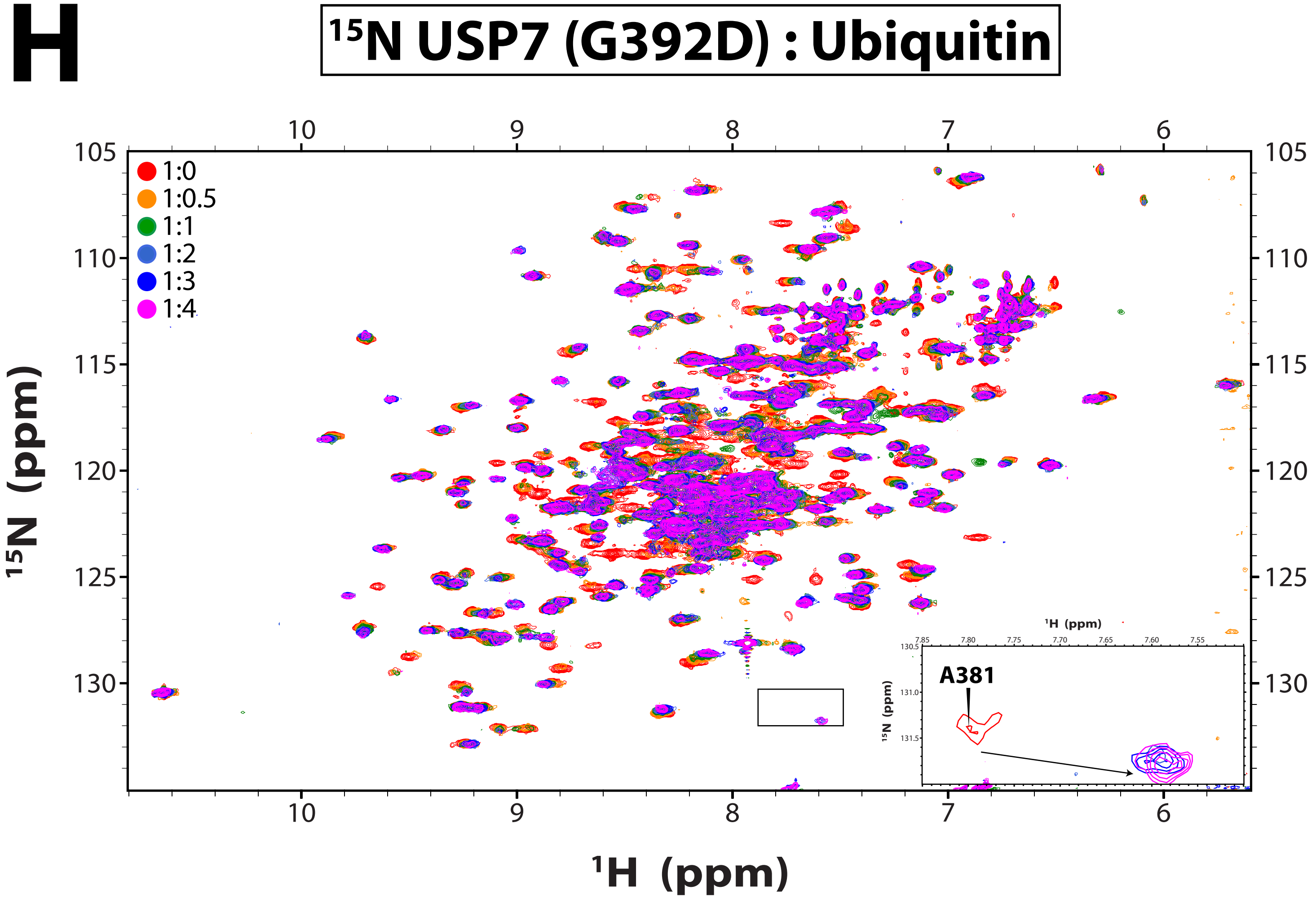

15N USP7 (G392D) : Ubiquitin
1:0
1:0.5
1:1
1:2
1:3
1:4
A381
15N (ppm)
1H (ppm)

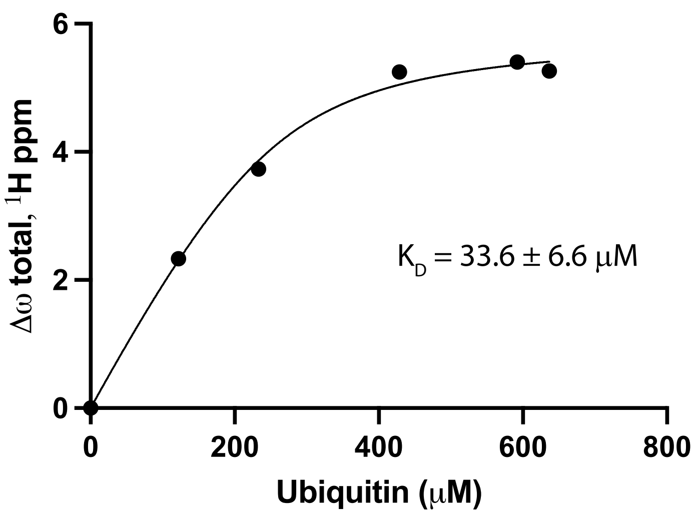

Δω total, 1H ppm
Ubiquitin (μM)
K_D = 33.6 ± 6.6 μM

Figure S4

I

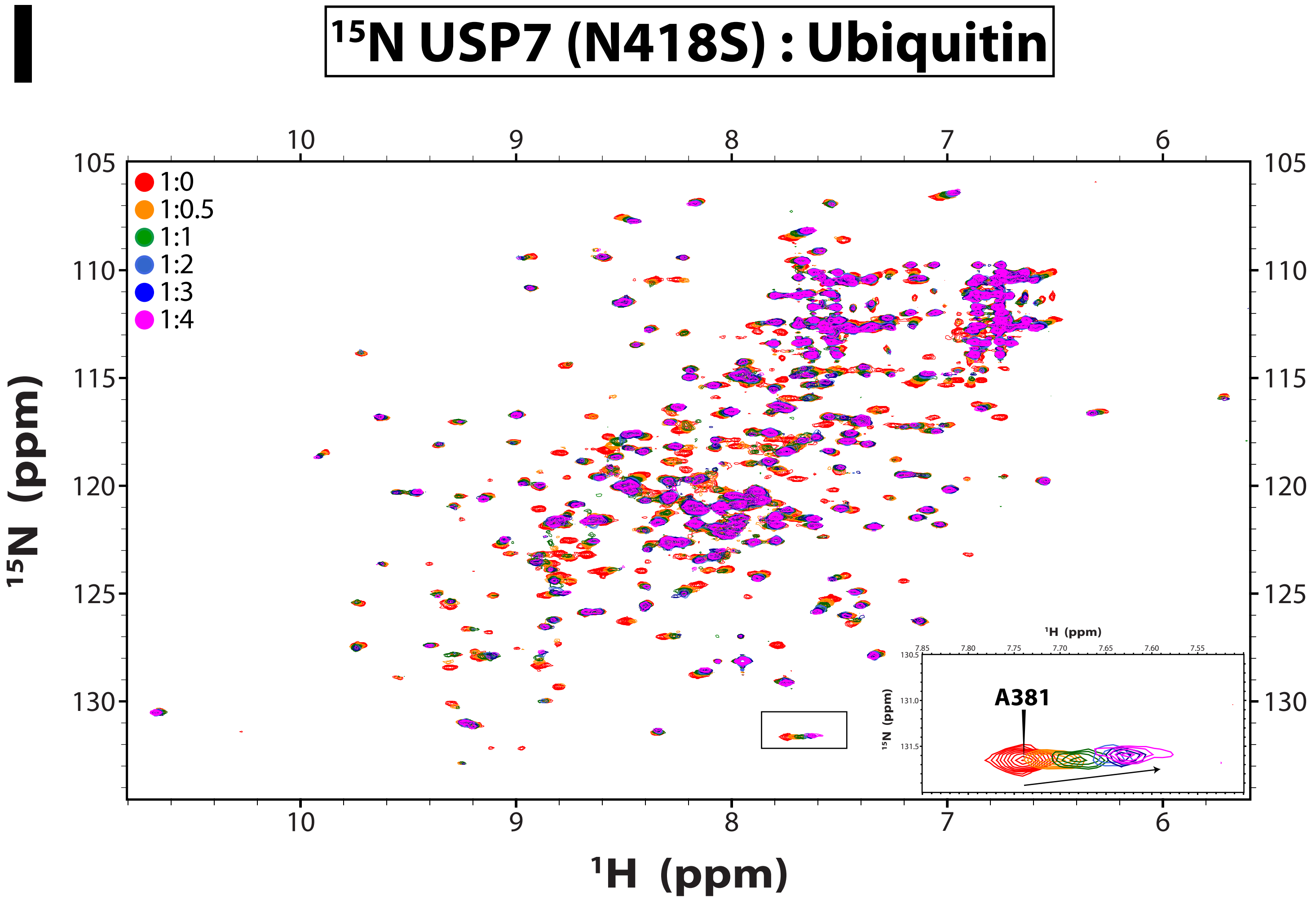

^{15}N USP7 (N418S) : Ubiquitin
1:0
1:0.5
1:1
1:2
1:3
1:4
A381
^{15}N (ppm)
^{1}H (ppm)

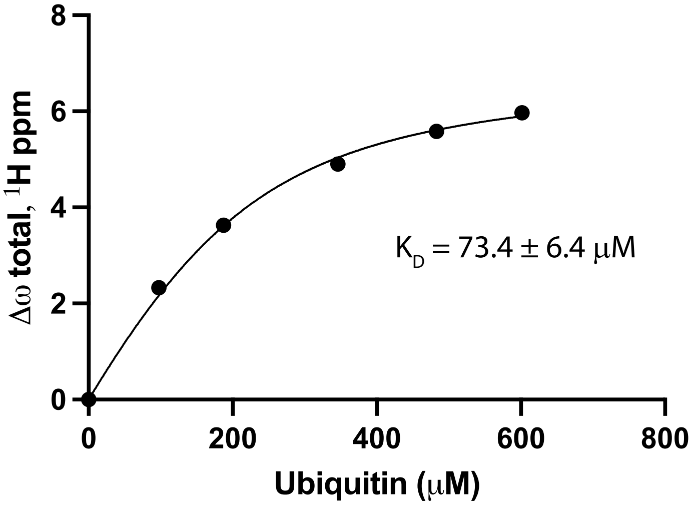

Δω total, ^{1}H ppm
Ubiquitin (μM)
K_D = 73.4 ± 6.4 μM

Figure S4

J

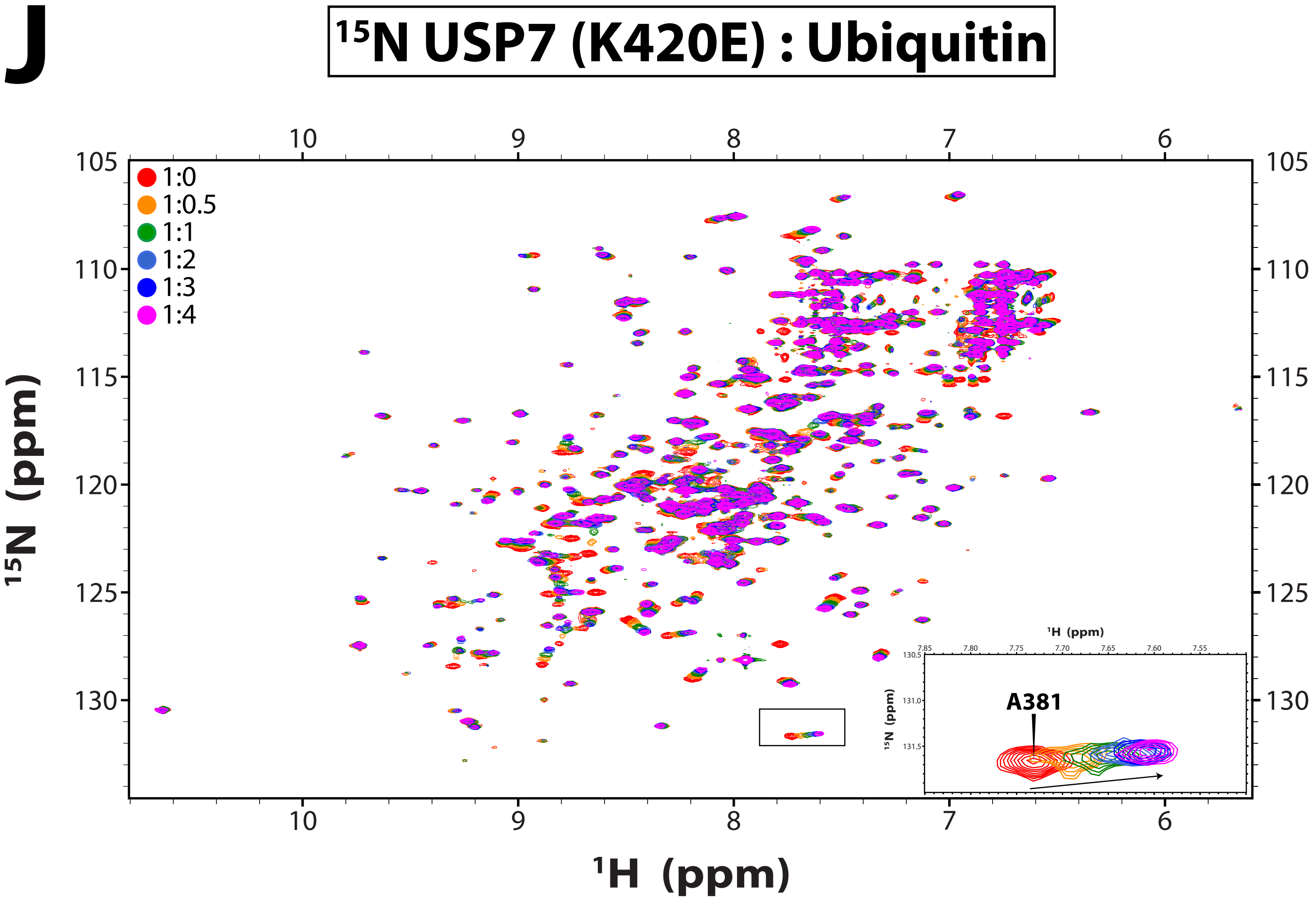

15N USP7 (K420E) : Ubiquitin
1:0
1:0.5
1:1
1:2
1:3
1:4
A381
15N (ppm)
1H (ppm)

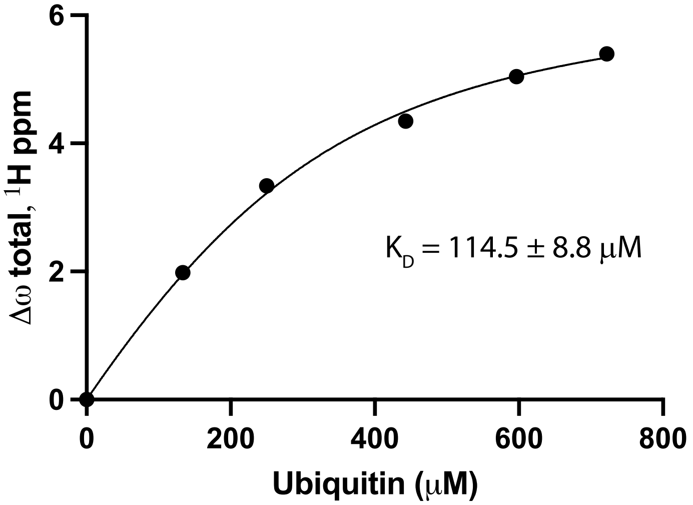

Δω total, 1H ppm
Ubiquitin (μM)
KD = 114.5 ± 8.8 μM

Figure S4

K

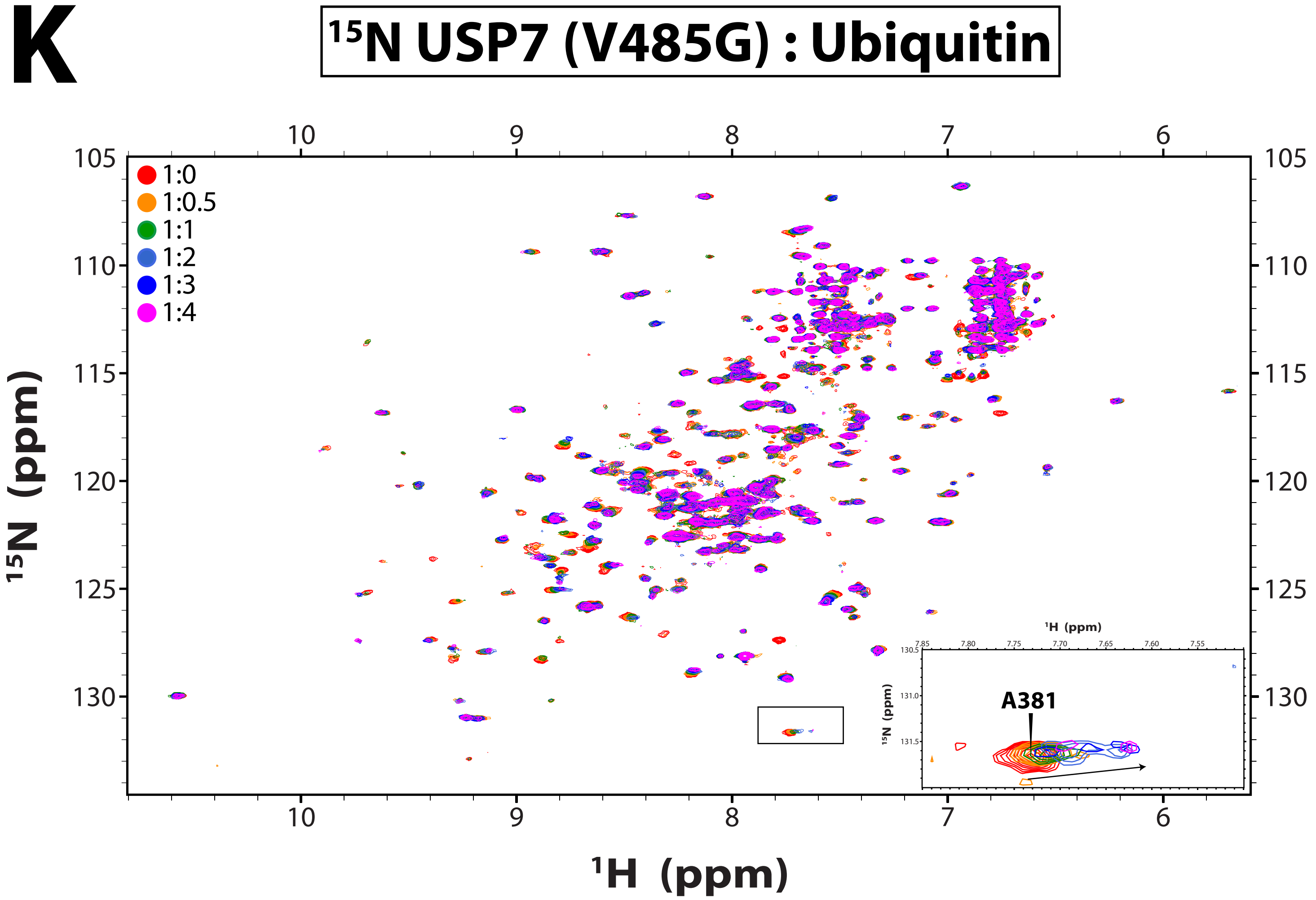

15N USP7 (V485G) : Ubiquitin
1:0
1:0.5
1:1
1:2
1:3
1:4
1H (ppm)
15N (ppm)
A381

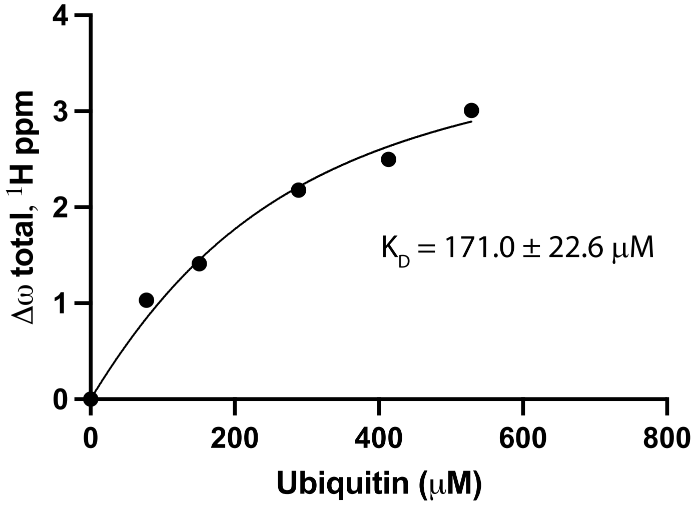

Δω total, 1H ppm
Ubiquitin (μM)
K_D = 171.0 ± 22.6 μM

Figure S4. Ubiquitin binding to catalytic domains of USP7.
Top: ^{15}N TROSY spectra of the $_{15}N$-labeled USP7 catalytic domain and its mutants gradually titrated with unlabeled ubiquitin. Residue A381 is showcased for each spectrum. USP7:ubiquitin molar ratios are shown. **Bottom:** Plot showing the global chemical shift perturbations ($\Delta\omega$ total) in the spectra, calculated as a sum of individual $\Delta\omega$ for all residues, as a function of ubiquitin concentration, used to estimate the binding affinities for each USP7 variant (K_D).

Figure S5

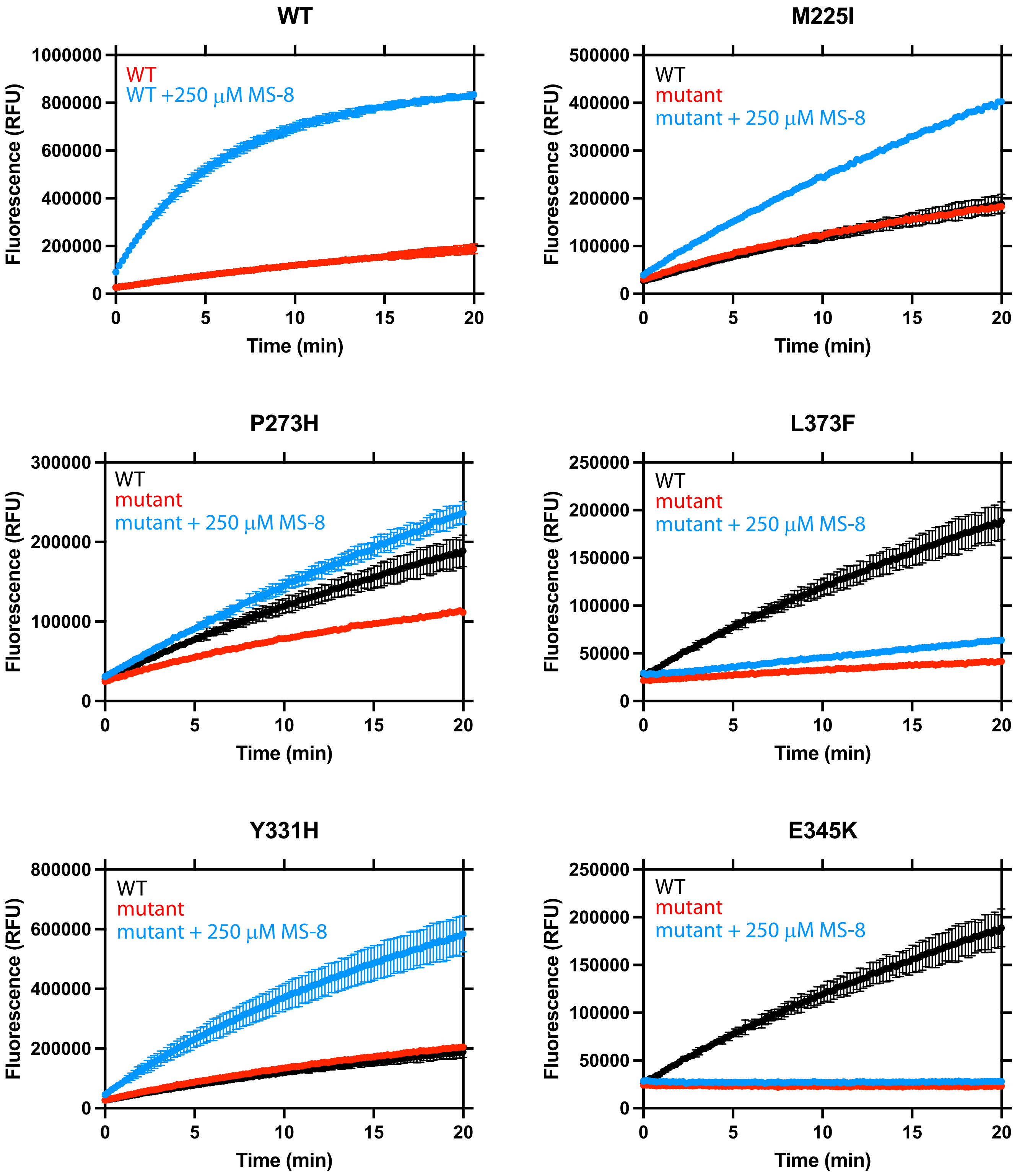
WT
Fluorescence (RFU)
Time (min)
WT
WT +250 μM MS-8
M225I
WT
mutant
mutant + 250 μM MS-8
P273H
L373F
Y331H
E345K

Figure S5

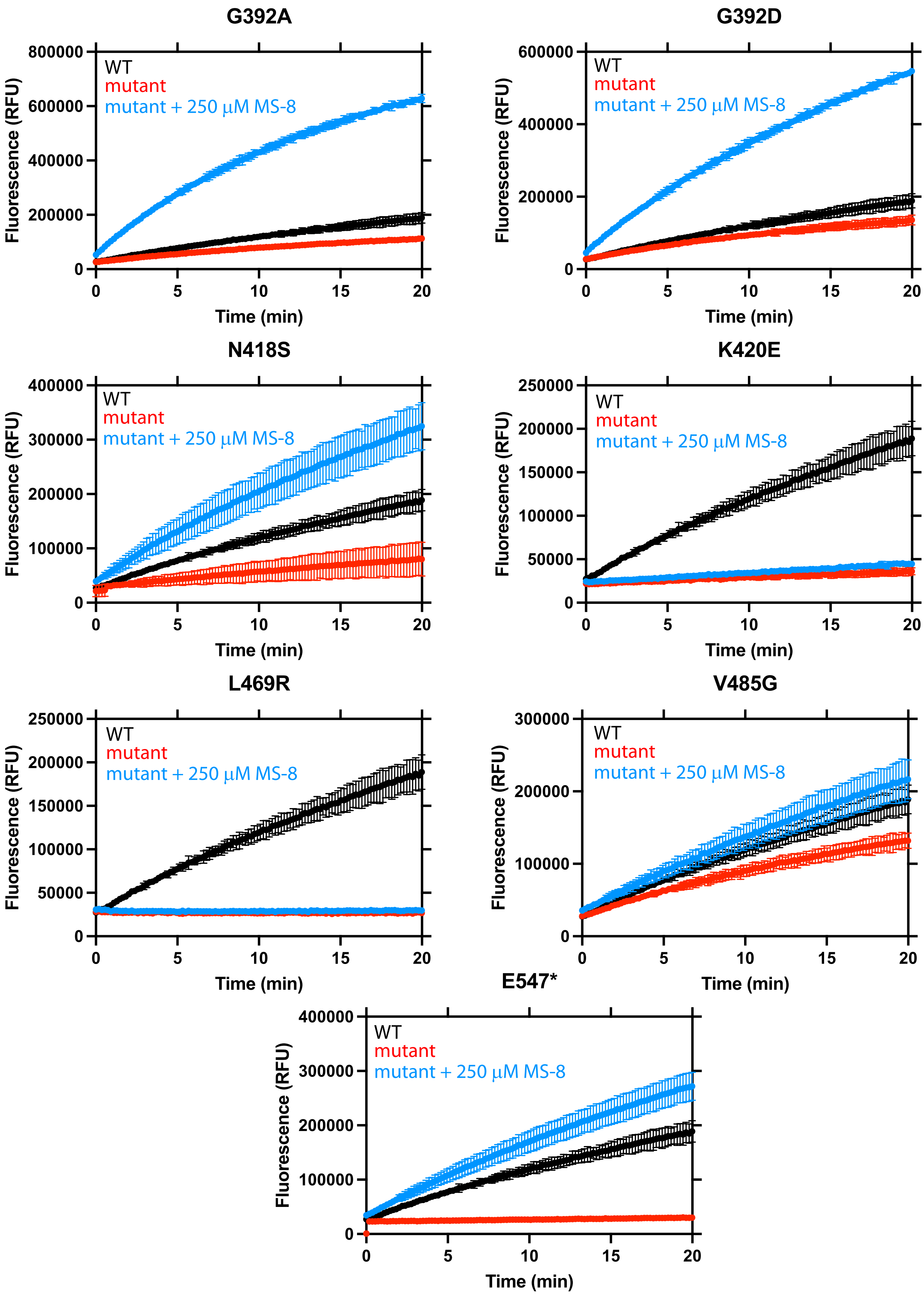

G392A
WT
mutant
mutant + 250 μM MS-8
Fluorescence (RFU)
800000
600000
400000
200000
0
0
5
10
15
20
Time (min)
G392D
WT
mutant
mutant + 250 μM MS-8
Fluorescence (RFU)
600000
400000
200000
0
0
5
10
15
20
Time (min)
N418S
WT
mutant
mutant + 250 μM MS-8
Fluorescence (RFU)
400000
300000
200000
100000
0
0
5
10
15
20
Time (min)
K420E
WT
mutant
mutant + 250 μM MS-8
Fluorescence (RFU)
250000
200000
150000
100000
50000
0
0
5
10
15
20
Time (min)
L469R
WT
mutant
mutant + 250 μM MS-8
Fluorescence (RFU)
250000
200000
150000
100000
50000
0
0
5
10
15
20
Time (min)
V485G
WT
mutant
mutant + 250 μM MS-8
Fluorescence (RFU)
300000
200000
100000
0
0
5
10
15
20
Time (min)
E547*
WT
mutant
mutant + 250 μM MS-8
Fluorescence (RFU)
400000
300000
200000
100000
0
0
5
10
15
20
Time (min)

Figure S5. MS-8 enhances the activity of USP7 variants.

Comparison of the time course of deubiquitination reaction for FL-USP7 variants alone (red) and treated with 250 μM MS-8 (blue). The untreated WT curve is shown for reference (black). 0.1 nM USP7 was used with 500 nM ubiquitin-rhodamine as its fluorogenic substrate.

Figure S6

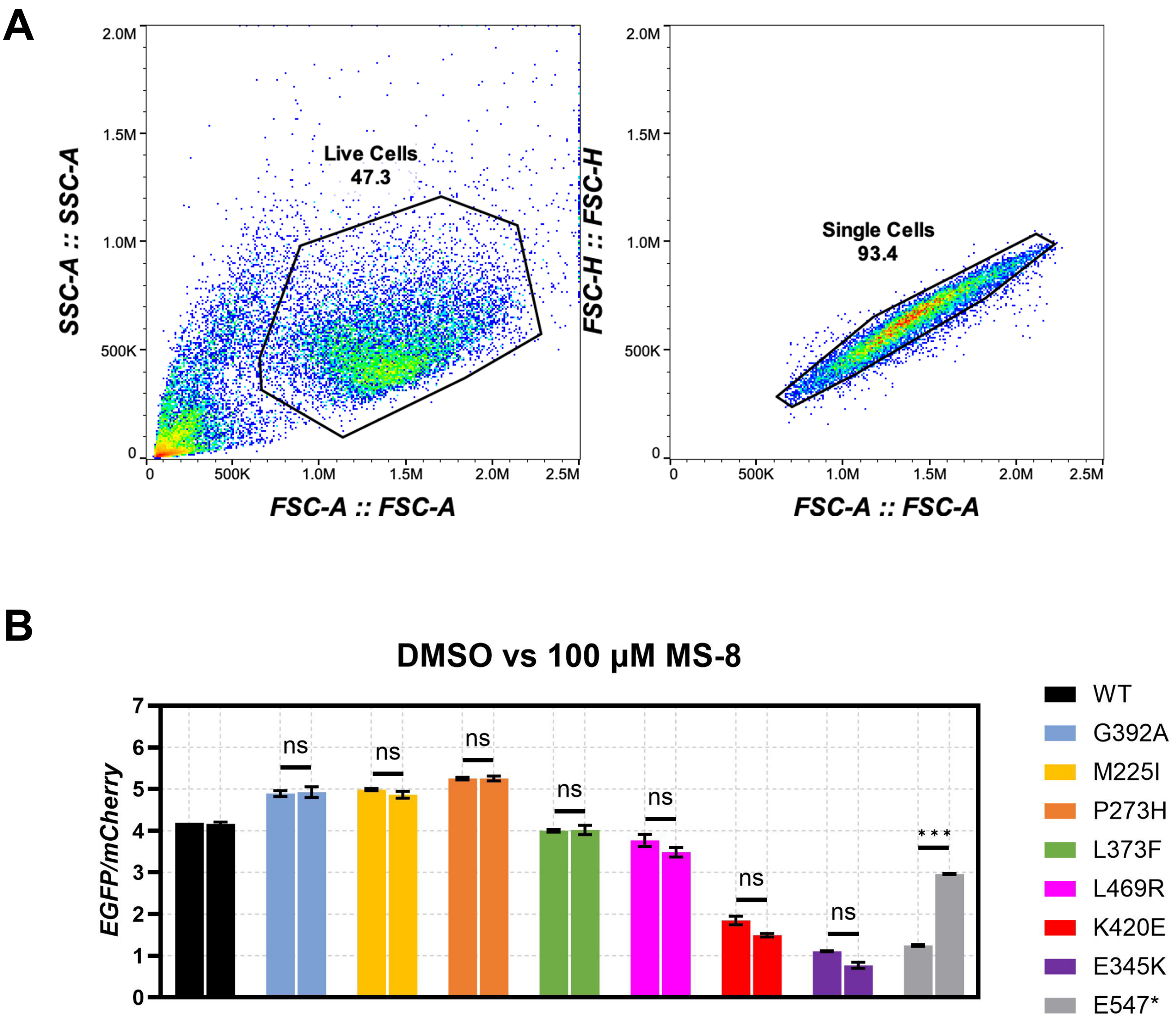

Figure S6.
A . Gating strategy conducted for USP7 analysis. 10,000 events were recorded in "Single Cells" gate for analysis. **B.** Comparison of USP7 DUB activity measured in Ubiquitin-GFP HEK293T cells expressing Hao-Fountain syndrome variants of USP7$^{cat\text{-}45}$ treated with either DMSO (left bar) or 100 mM MS-8 (right bar).

Figure S7

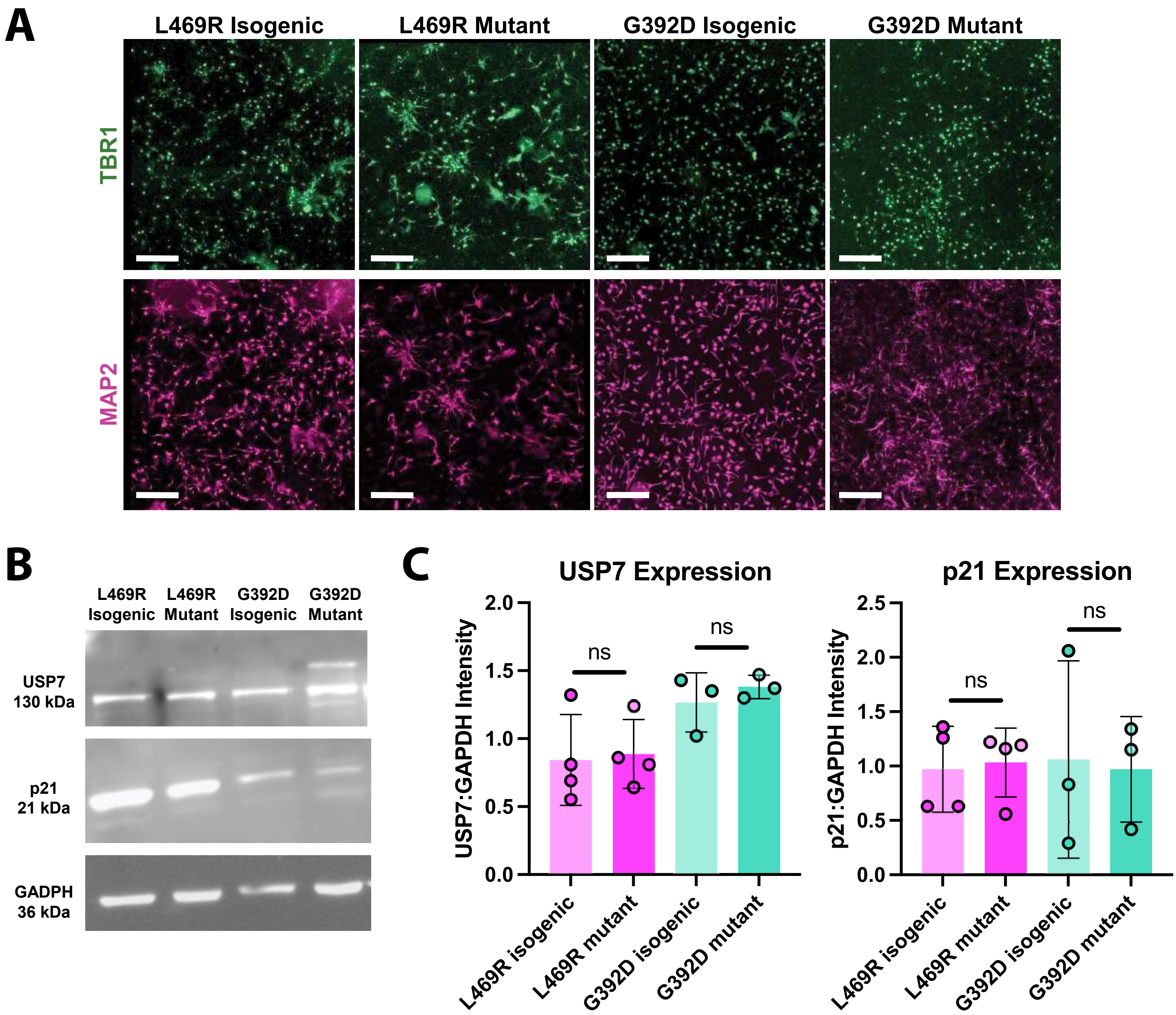
A
L469R Isogenic
L469R Mutant
G392D Isogenic
G392D Mutant
TBR1
MAP2
B
L469R Isogenic
L469R Mutant
G392D Isogenic
G392D Mutant
USP7
130 kDa
p21
21 kDa
GADPH
36 kDa
C
USP7 Expression
USP7:GAPDH Intensity
2.0
1.5
1.0
0.5
0.0
ns
ns
L469R isogenic
L469R mutant
G392D isogenic
G392D mutant
p21 Expression
p21:GAPDH Intensity
2.5
2.0
1.5
1.0
0.5
0.0
ns
ns
L469R isogenic
L469R mutant
G392D isogenic
G392D mutant

Figure S7. Hao-Fountain patient derived induced pluripotent stem cells (iPSC) are capable of making cortical neurons and express USP7 and p21.

A. Representative immunofluorescent images of cortical neurons (Tbr1+/MAP2+) derived from Hao-Fountain iPSC and their isogenic (corrected) controls. Tbr1 is a cortical layer IV marker while MAP2 is a neuronal specific microtubule marker. The scale bar represents 200 µm. **B.** Representative Western blot of cortical neuron lysates for USP7, USP7 substrate p21, and the loading control GAPDH. **C.** Western blot quantification of USP7 and p21 expression in cortical neuron lysates. L469R pair n = 4 independent differentiations, G392D pair n = 3 independent differentiations. Significance was calculated via a Mann-Whitney nonparametric test. ns = not significant.

Table S1
Summary of enzyme kinetics and ubiquitin-binding affinities of USP7 mutations associated with Hao-Fountain syndrome.

	WT	M225I	P273H	Y331H	E345K	L373F	G392A	G392D	N418S	K420E	L469R	V485G
**Catalytic domain**												
k_{cat} (min^{-1})	1.6±0.0	2.5±0.2	1.3± 0.2	2.4±0.1	0.1±0.0	0.8±0.0	3.6±0.2	3.5±0.1	10.6±0.7	N/A	0.5±0.0	0.2±0.0
K_M (μM)	0.6± 0.1	0.6±0.1	1.9±0.4	0.1±0.1	N/A	1.1± 0.2	0.2±0.1	0.1±0.0	0.3±0.1	N/A	0.2± 0.1	N/A
k_{cat}/K_M ($min^{-1}\mu M^{-1}$)	2.9±0.3	4.6±1.1	0.7±0.2	18.6±7.0	N/A	0.7±0.1	17.8±4.8	35.9±11.4	30.9±9.2	N/A	2.9±1.1	N/A
K_D (μM)	160.1±24.2	91.6±5.2	82.5±9.9	26.0±7.9	621.0±56.6	107.9±29.8	77.2±4.7	33.6±6.6	73.4±6.4	114.5±8.8	N/A	171.0±22.6
**Full-length**												
k_{cat} (min^{-1})	56.2±3.4	25.5± 2.7	19.7±0.7	45.3±3.4	0.2± 0.0	16.1±3.1	31.9±1.1	30.9±1.5	16.1±0.7	1.0±0.1	1.8±0.2	1.3±0.1
K_M (μM)	0.7±0.1	0.5±0.1	0.2±0.0	0.6±0.1	N/A	2.8±0.8	0.3±0.0	0.3±0.0	0.2±0.0	N/A	0.21±0.1	N/A
k_{cat}/K_M ($min^{-1}\mu M^{-1}$)	86.3±14.4	55.1±16.4	132.0±20.8	76.0±15.9	N/A	5.9±2.0	104±13.1	123.1±21.6	102.5±20.2	N/A	8.2±3.0	N/A

Supplementary Material and Methods

Patient induced pluripotent stem cell (iPSC) maintenance and differentiation:

Hao-Fountain patient iPSC lines were obtained from CombinedBrain with their isogenic (mutation corrected) match. Pluripotency was confirmed with immunofluorescent labeling of SSEA4 (1:100, BD Pharmingen™, Cat# 560308) and SOX2 (1:200, ThermoFisher Scientific, Cat# MA1-014) along with gross colony morphology. iPSCs were maintained on hESC qualified Matrigel (Corning) coated dishes with mTeSR Plus (Stem Cell Technologies) in a 5% CO_2 37°C humidified incubator. When iPSC reached ~75% confluency, they were passaged as colonies with 0.5 mM EDTA and passages were kept to 10 or less for an independent thaw.

iPSC were grown to ~90% confluency and then dissociated to single cells using Accutase (Corning). Single cells were plated at 5 x 10^6 cells per mL in 5 mL of mTeSR Plus with 10µM ROCK inhibitor (Y-27632) in each well of an untreated (suspension) 6-well plate. Plates were rocked at 120 rpm inside the incubator to form embroid bodies and then differentiated to cortical neurons using an established protocol(1). At day 35 of the differentiation protocol, neurons were dissociated to single cells using Accutase and plated in 96 well plates coated with poly-ornithine, poly-D lysine, laminin and fibronectin with 10µM ROCK inhibitor as previously described(2). The next day, the ROCK inhibitor was removed, and fresh maturation basal media was added (Neurobasal A media, 1X pen/strep, 1X Glutamax, 1X NEAA, 1X B27, 100µM 2-mercaptoethanol, 20 ng/ml BDNF, 20 ng/ml GDNF, 20 ng/ml NT3, 20 ng/ml NGF and 50 µM L-ascorbic acid). Cortical neurons were grown for 2 more days before analysis (total of 72 hours post dissociation/plating). Cortical neuron identity was confirmed with immunofluorescent staining for the cortical transcription factors Tbr1, SATB2, CTIP2 and the neurite specific cytoskeletal marker MAP2.

Western Blot:

On day 35 of the differentiation protocol, an aliquot of the cortical spheroids was washed with phosphate buffer saline (PBS) and then lysed with RIPA buffer containing protease inhibitor (HALT Protease, Pierce) and DNase. Lysates were treated with 50µM PR-619 DUB inhibitor (Selleckchem, cat# S7130) and then centrifuged at 16,000 x g for 5 minutes at 4°C. The protein mixture was separated into a supernatant and a cell pellet. The supernatant (lysate) was then used as the source of protein. The protein lysate was then quantified using the Pierce™ Dilution-Free™ Rapid Gold BCA Protein Assay Kit (ThermoFisher Scientific, Cat# A55860). 20 µg of each protein lysate was then loaded onto an SDS polyacrylamide gel (12% resolving and 4% stacking gel) along with a pre-stained protein ladder and subjected to electrophoresis. The samples were transferred onto a PVDF membrane using a Transblot Turbo (BioRad). When the transfer was completed, the membrane was blocked with 5% fat free milk in 1X phosphate buffer saline with 0.1% Tween (1X PBST) for 30 minutes. The blocked membrane was then incubated at 4°C overnight with a mix of primary antibodies (USP7 1:1000, Cell Signaling Technology, cat# 4833S; GAPDH 1:5000, Proteintech, cat# 60004-1-Ig; p21 1:1000, Cell Signaling Technology, cat# 2947S). The next day, the membrane was washed with PBST and then incubated with HRP-conjugated secondary antibodies (Goat Anti-Rabbit 1:2000 ThermoFisher Scientific cat# 31450, Goat Anti-Mouse 1:2000 ThermoFisher Scientific cat# 31430) for 45 minutes. After the incubation, the membrane was washed again with PBST. The membrane was then covered with a mixture of SuperSignal™ West Pico PLUS Chemiluminescent Substrate (ThermoFisher Scientific, cat# 34577) and imaged using the Syngene™ G:Box mini 6 imaging system. Western blots were quantified using the ImageJ. Band intensities were normalized to the GAPDH signal for each sample. Each data point represents a sample from an independent differentiation (i.e. biological replicate). Statistical analysis was performed in GraphPad Prism (version 10.4.0) using a two-tailed, non-parametric, Mann-Whitney Test.

Immunofluorescent Labeling and Image Analysis:

Three days after plating, cortical neurons were fixed with 4% paraformaldehyde, washed and blocked, and then treated with primary antibodies at 4°C overnight (MAP2 1:1000, ThermoFisher Scientific, cat# PA1-16751; Tbr1 1:100, Abcam Cat# ab31940). The next day, plates were washed with phosphate buffer saline (PBS), and Alexa-conjugated secondary antibodies (1:1000 ThermoFisher Scientific) along

with Hoescht (1:5000, ThermoFisher Scientific, Cat# 62249) were added for 45 minutes at room temperature. Plates were washed once more and then imaged in PBS on a Molecular Devices Pico High Content Imager. For cortical neuron identity, images were analyzed in CellReporterXpress (Molecular Devices) for overlap of nuclei (Hoechst) and the individual cortical transcription factor (Tbr1). A differentiation was considered successful if it had at least 75% neural identity. Images were imported into MetaXpress (Molecular Devices) for analysis. Immunofluorescent signals three times the intensity of background were considered positive.

Supplementary References

1. D. Whye *et al.*, A Robust Pipeline for the Multi-Stage Accelerated Differentiation of Functional 3D Cortical Organoids from Human Pluripotent Stem Cells. *Curr Protoc* **3**, e641 (2023).
2. D. Kulick *et al.*, Amyotrophic Lateral Sclerosis-Associated Persistent Organic Pollutant cis-Chlordane Causes GABA(A)-Independent Toxicity to Motor Neurons, Providing Evidence toward an Environmental Component of Sporadic Amyotrophic Lateral Sclerosis. *ACS Chem Neurosci* **13**, 3567-3577 (2022).
